# Intronic SNP in *ESR1 encoding* human estrogen receptor alpha is associated with brain ESR1 mRNA isoform expression and behavioral traits

**DOI:** 10.1371/journal.pone.0179020

**Published:** 2017-06-15

**Authors:** Julia K. Pinsonneault, John T. Frater, Benjamin Kompa, Roshan Mascarenhas, Danxin Wang, Wolfgang Sadee

**Affiliations:** Center for Pharmacogenomics, Department of Cancer Biology and Genetics, College of Medicine and Public Health, Ohio State University, Columbus, Ohio, United States of America; National Cancer Institute, UNITED STATES

## Abstract

Genetic variants of *ESR1* have been implicated in multiple diseases, including behavioral disorders, but causative variants remain uncertain. We have searched for regulatory variants affecting ESR1 expression in human brain, measuring allelic ESR1 mRNA expression in human brain tissues with marker SNPs in exon4 representing ESR1-008 (or ESRα-36), and in the 3’UTR of ESR1-203, two main ESR1 isoforms in brain. In prefrontal cortex from subjects with bipolar disorder, schizophrenia, and controls (n = 35 each; Stanley Foundation brain bank), allelic ESR1 mRNA ratios deviated from unity up to tenfold at the exon4 marker SNP, with large allelic ratios observed primarily in bipolar and schizophrenic subjects. SNP scanning and targeted sequencing identified rs2144025, associated with large allelic mRNA ratios (p = 1.6E10^-6^). Moreover, rs2144025 was significantly associated with ESR1 mRNA levels in the Brain eQTL Almanac and in brain regions in the Genotype-Tissue Expression project. In four GWAS cohorts, rs2104425 was significantly associated with behavioral traits, including: hypomanic episodes in female bipolar disorder subjects (GAIN bipolar disorder study; p = 0.0004), comorbid psychological symptoms in both males and females with attention deficit hyperactivity disorder (GAIN ADHD, p = 0.00002), psychological diagnoses in female children (eMERGE study of childhood health, subject age ≥9, p = 0.0009), and traits in schizophrenia (*e*.*g*., grandiose delusions, GAIN schizophrenia, p = 0.0004). The first common *ESR1* variant (MAF 12–33% across races) linked to regulatory functions, rs2144025 appears conditionally to affect ESR1 mRNA expression in the brain and modulate traits in behavioral disorders.

## Introduction

The estrogen signaling pathway plays a pervasive role throughout the body. Significant evidence supports a role in neuropsychiatric disorders, modulating positive symptoms of schizophrenia, mania in bipolar individuals, and cognition and emotion as a result of hormonal fluctuations [1]. Most studies on *ESR1* have focused on effects observed in females; however, some estrogen receptor isoforms appear to signal in a ligand-independent fashion [2, 3] and estrogens are generated in the brains of females and males. Therefore, signaling via estrogen receptor alpha (*ESR1*) is critical not only in females but also in males [4]; yet, few studies on *ESR1* have focused on males, mostly with inconclusive results [5, 6]. Uncertainty in defining causative genetic variants has confounded interpretation of these clinical studies. While *ESR1* had not shown robust associations with a diagnosis of psychiatric diseases in case-control studies, numerous studies have identified significant associations between *ESR1* variants and behavioral abnormalities and traits[6–9]; (Tables A & B in S1 File). A role of *ESR1 variants* in behaviors is further supported by animal studies, for example mating behavior in zebra finches [10] and social behaviors in sparrows [11]. Therefore, we focus here on *ESR1* variants affecting behavioral traits in neuropsychiatric disorders, including bipolar disorder, ADHD, and schizophrenia [12].

As non-synonymous SNPs are rare in *ESR1*, polymorphisms affecting gene expression and RNA processing may account for reported behavioral symptoms associated with *ESR1*, a mechanism consistent with the high prevalence of non-coding variants in GWAS results and molecular genetics studies [13–21]. Multiple non-coding *ESR1* variants have been implicated as candidates affecting mRNA levels (expression quantitative trait loci; eQTLs) [13] and behavioral traits [1, 6–9, 22–28]. (Table A in S1 File). Significant associations typically are contingent upon sex, age, hormonal fluctuations, puberty, menopause, and diagnosis [1, 14–21]. However, a complex 450kb gene structure impedes discovery of causative regulatory variants in *ESR1*, with multiple promoters and transcription start sites that regulate expression of 8 non-coding exons upstream of the 8 coding exons. Moreover, distinct 3’UTRs and alternative splicing events generate additional ESR1 RNA isoforms [2], each with potentially distinct functions. Expression profiles of the ESR1 mRNA isoforms vary between tissues, so that regulatory variants likely have distinct effects in different tissue. In addition, the ESR1 mRNA isoform profiles can change with disease state, for example in schizophrenia, bipolar disorder, and depression, where the canonical full-length form of ESR1 mRNA is reduced or becomes undetectable within the brain [12]. In searching for functional variants, we therefore have to consider the target tissue (brain regions in this study), sex, age, and psychiatric disorders, to reflect possible dynamic interactions with regulatory variants on ESR1 expression.

The aim of this study was to discover regulatory *ESR1* variants, in a first step employing allelic expression imbalance (AEI) in brain autopsy tissues as a sensitive indicator of *cis-*acting variants affecting either transcription or RNA functions[29]. Together with genotype information at the level of gDNA, AEI ratios serve as markers to scan a gene locus for causative regulatory variants. Allelic mRNA ratio analysis is more sensitive and accurate than considering eQTLs associated with total mRNA levels[29]. Since regulation of *ESR1* expression appears to be contingent on multiple factors and disease states[1, 14–21], we measured AEI in brain tissues from subjects diagnosed with schizophrenia and bipolar disorder, and from controls, using marker SNPs in expressed RNA isoforms. Allelic expression imbalance of ESR1 mRNA was most pronounced in brain tissues from subjects with bipolar disorder and schizophrenia compared to controls, suggesting regulatory processes that change with disease status as a function of *trans-*acting factors, as observed previously[24, 25]. SNP scanning identified a single strong candidate regulatory variant (rs2144025) located in intron4, associated with expression of ESR1 mRNA isoforms. Lastly, we evaluated the effect of rs2144025 in four GWAS, with a focus on behavioral traits in psychiatric disorders, controlling for sex and age of the subjects. The results support the hypothesis that rs2144025 is associated with both expression and clinical traits.

## Results

### Clinical association of *ESR1* SNPs in the literature

A PubMed literature search was conducted compiling every SNP with available clinical associations, for selection of candidate SNPs in this study. Table A in S1 File lists over 60 *ESR1* SNP variants, sorted by genomic position, showing significant clinical associations with six phenotypic categories in candidate gene studies: Bone/Joint, Cancer, Cardiovascular, CNS, Infection, and Fertility. Variants with multiple associations are located in intron1: rs2234693 and rs9340799. Since many of the SNPs with significant associations are not in high LD with each other, these results suggest a complex landscape of functionally relevant polymorphisms, while the causative variants have remained elusive. Table B in S1 File summarizes significant GWAS hits attributable to the *ESR1* locus, associated with a broad spectrum of traits and disorders, consistent with the pervasive role of estrogens throughout the human body. In this study, we will focus on CNS-related traits and behaviors.

### Expression of ESR1 mRNA isoforms in GTEx and quantitative RT-PCR analysis in human tissues

**Fig 1:**
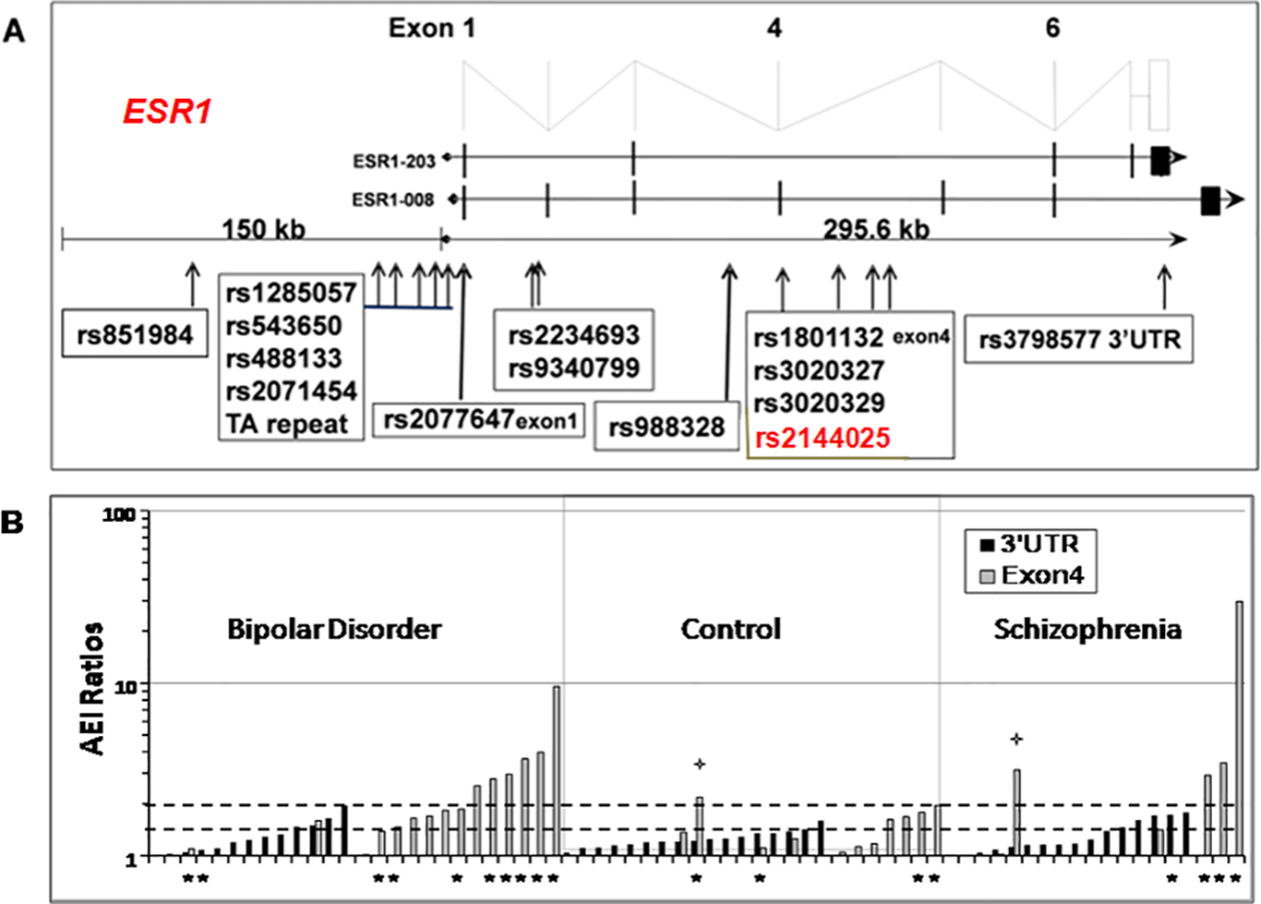
***ESR1* gene structure, mRNA isoforms and variants (A), and allelic mRNA ratios in Stanley PFC brain samples(B).** A) Estrogen receptor α gene map showing exon structure, location of genotyped SNPs, and the mRNA isoforms ESR1-203 and ESR1-008. The 150 kb upstream region contains multiple alternative promoters and first exons. With deep sequencing, additional exons have been added to the *ESR1* locus, in particular within the 150 kb 5’ region; numbering of the exons here reflects historical usage. B) Absolute allelic ratios measured in 70 Stanley PFC samples using 2 marker SNPs, sorted by disease status. *subjects heterozygous for rs2144025. Dashed lines: cut off 2.0 for high AEI ratios and 1.4 for moderate AEI. ^+^2 samples with discrepant allelic ratios from the two marker SNPs were counted as moderate AEI. None of the subjects with moderate and high AEI were in the control group.rs2144025is in low LD with the two marker SNPs rs3798577 (R^2^ = 0.003) and rs1801132 (R^2^ = 0.044) in the CEU(1000 genomes) cohort; therefore, only absolute AEI ratios are shown.

Annotated ESR1 mRNA isoforms from Ensembl (ensembl.org) show multiple transcription start sites, alternative splicing events, and variable 3’UTRs (Fig 1). From transcriptome RNAseq data provided in GTEx (http://www.gtexportal.org/home/), we obtained estimated relative distributions of the mRNA isoforms across human tissues (Fig 1 and Fig A in S1 File).In brain tissues, two main transcripts appear to prevail, ESR1-203 and ESR1-008(which is also termed ESRα-36), the latter more highly expressed except in the hypothalamus. While these two isoforms identified by RNA sequencing data in GTEx are likely the main components, other isoforms may not have been detectable with RNAseq (for example the canonical ESR1 with 8 coding exons), as a result of limitations in transcript assignments.ESR1-203 appears not to express a functional estrogen receptor, lacking key elements of estrogen and DNA interaction sites, leaving its biological function uncertain. On the other hand, ESR1-008 has been described as signaling in an estrogen-dependent and independent fashion[3]. ESR1-008contains a unique 3’UTR exon further downstream and differs from ESR1-203 by inclusion of exon4, while ESR1-203 contains a more proximate 3’UTR shared with other isoforms (Fig 1).

Relative expression of ESR1 mRNA was determined by qRT-PCR in multiple samples from prefrontal cortex and putamen, with primers targeting the 3’UTR of ESR1-203 and exon4 of ESR1-008. Cycle thresholds ranged between 24–28, showing that both isoforms were detectable at levels sufficient for accurate allelic mRNA ratio analysis (Ct < 30). mRNA levels of both isoforms measured in autopsy brain regions of 105 subjects diagnosed with bipolar disorder (BD) and schizophrenia, and controls (n = 35 each, from the Stanley Foundation) were not significantly different between the three groups.

### Analysis of allelic mRNA expression imbalance (AEI) for detection of regulatory variants

We selected two marker SNPs for allelic ratio analysis, rs1801132 in exon4 representing ESR1-008, and rs3798577 in the 3’UTR exon representing ESR1-203. This approach approximates measurements of allelic ratios of these two mRNA isoforms in brain tissues, whereas in other tissues the predominance of additional isoforms would confound the results (Figure A in S1 File).In a first series of experiments, we observed allelic mRNA ratios deviating from unity (normalized to gDNA ratios), demonstrating moderate allelic expression imbalance indicative of regulatory variants in breast tissues and in autopsy brain tissues (putamen and prefrontal cortex) from subjects without a diagnosis of mental disorders (Figure B in S1 File). We were unable to link these to candidate regulatory variants affecting expression of ESR1 mRNA or any of its isoforms.

We subsequently turned to RNA and DNA samples extracted from autopsy brain regions of a cohort of105 subjects diagnosed with bipolar disorders, schizophrenia, and controls (Stanley Foundation).Allelic ratios of mRNA and genomic DNA (major/minor allele) were measured in 46 PFC tissues heterozygous for marker rs3798577 in the 3’UTR (representing mainly ESR1-203), and in 33 samples heterozygous for rs1801132 in Exon4 (representing mainly ESR1-008), with 9 tissues heterozygous for both, showing that the marker SNPs were in low LD between each other (R^2^ and D’ = 0.00).Copy number variants yielding allelic ratios deviating from unity were not detected in genomic DNA. We define presence of AEI as deviation of allelic mRNA ratios more than 3xSD from the mean in cDNA repeats, after correction for allelic gDNA ratios; this threshold was set at 1.37 for rs3798577 and at 1.38 for rs1801132.Allelic expression measured with both markers displayed frequent AEI with ratios evenly distributed in both directions <1 and >1, indicating that any regulatory variants accounting for the AEI ratios are not in LD with the marker SNPs.Fig 1B displays absolute AEI ratios, regardless of measured major/minor allele ratios <1 or>1. Large AEI ratios up to 12-fold were observed only with marker rs1801132 in exon4 (ESR1-008). In the 7 subjects heterozygous for both markers, the AEI ratios were similar but diverged in 2 subjects, likely attributable to different distributions of the two primary mRNA isoforms [22].

To scan the gene locus for causative variants in heterozygous individuals when AEI is detected, we first genotyped 11 candidate variants (Fig 1A), some previously associated with multiple clinical phenotypes including a *TA* repeat (rs71685044) associated with postpartum depression [14] (Table A in S1 File), in all 70 tissues heterozygous for either or both marker SNPs (Table 1). Linkage disequilibrium (LD) between each variant analyzed is provided in Table C in S1 File, showing that the selected SNPs cover several haplotype blocks in *ESR1*.Use of all significant AEI ratio (>1.37–1.38) failed to reveal strong associations with any candidateSNP; therefore we focused on large AEI ratios >2, observed only in the bipolar and schizophrenic subjects with rs1801132 exon4 marker. High AEI ratios >2 were found in 6 bipolar and 3 schizophrenia subjects but not in the controls. The association with diagnosis was significant at p = 0.05 forBP *versus* controls, with a similar trend observed in schizophrenia (Table 2 and Fig 1B). The association of large AEI ratios with disease status supports the hypothesis that a regulatory variant may be sensitive to environmental or pathophysiological conditions.

**Table 1:** Association of *ESR1* variants with large allelic expression imbalance (ratios >2) in the Stanley prefrontal cortex brain samples.

Marker	Minor allele frequency %	F-TestP	F-Test Bonf. P
rs851984	38.5	0.07	0.87
rs1285057	37.4	0.52	1
rs543650	37.4	0.80	1
rs488133	31.2	0.71	1
rs2071454	12.2	0.09	1
rs71685044*	45.6	0.92	1
rs2077647	50.0	0.20	1
rs2234693	49.0	0.95	1
rs9340799	36.1	0.41	1
rs988328	14.7	0.001	0.02
rs3020327	10.9	0.09	1
rs3020329	27.6	0.007	0.08
rs2144025	18.1	1.58E-06	2.05E-05

All variants were genotyped in all 70 brain tissues heterozygous for a marker SNPs, including SNPs identified after full sequencing of 5’ regions and the large intron4 in 9 tissues.AEI ratios were measured at two marker SNPs, rs1801132 and rs3798577 (Fig 1). The ratios were categorized into high AEI (ratios >2), moderate AEI (ratios >1.3≤1.9) or no AEI (ratios < 1.3). Associations between each variant and AEI categories were determined. High expressing allele/low expressing allele AEI ratio values were used since the marker SNPs did not appear to be in LD with a regulatory variant causing AEI (see legend Fig 1)and all genotypes were coded as heterozygous or homozygous.

*TA repeat

**Table 2:** Distribution of AEI ratio category versus diagnosis.

AEI Category	rs2144025			Diagnosis		
	CC	CT	TT	Control	BD	Schizo.
0	30	3	2	17	10	8
1	14	6	2	6	9	7
2	2	9	0	1	7	3

**0**: no AEI; **1**: intermediate AEI ratios 1.4–2; **2**: ratios >2 fold. Strong AEI ratios>2 were significantly associated with bipolar disorder (Fisher’s exact p = 0.05 in a 2x3 contingency table) and trended towards association with schizophrenic subjects.

### Targeted sequencing and genotyping of the ESR1 locus

Associations of large AEI ratios observed with marker rs1801132 pointed to two regions carrying candidate variants, exon4-intron4-exon5 (~80 kb) and nine regions covering the 5’ alternative untranslated exons spread over 150 kb. These were sequenced in 9 samples (4 with large AEI ratios and 5 without AEI). In the 5’ region, only rs851984 correlated with AEI (p = 0.007, Table D in S1 File). The second region including the 67kb intron4, previously implicated in association studies [16, 23, 26–28, 30–36], was completely sequenced, yielding 140 SNPs (Table D in S1 File), and a second candidate SNP, rs2144025, associated with AEI (p = 0.007): all 4 samples with large AEI were heterozygous, while 4 out of 5 subjects without AEI were homozygous.

We then genotyped both rs851984 and rs2144025, together with other candidate SNPs, in all 70 tissues with AEI ratios (Table 1). By far the most significant SNP to account for the large AEI ratios was rs2144025 (p = 1.6E-06). Analysis of the 1,000 genomes project indicated that rs2144025 is in moderate LD with other variants in *ESR1* implicated in clinical association studies (Table I in S1 File), but these were unlikely to account for the observed AEI pattern because of low MAF. AEI ratios associated with rs2144025 vary over a large range, indicative of a regulatory mechanism sensitive to *trans*-acting factors, as observed for an intronic SNP affecting CYP3A4 mRNA expression (range 1.6–6 fold) [37].These results with AEI ratios suggest that rs2144025 is a strong candidate variant with robust effect on mRNA levels in the PFC, while *ESR1* likely harbors additional regulatory variants not accounted for in this study.

#### Associations of ESR1 rs2144025 with mRNA levels

We used poly-*dT* plus targeted (exon4 and 3’UTR) primers to measure ESR1-203 and ESR1-008 in prefrontal cortex autopsy tissues from the Stanley Foundation. The results are shown in Figure C in S1 File, grouped into bipolar, schizophrenia, and control subjects, and by genotype status of rs2144025. While differences in mRNA isoforms levels were not significant between controls and affected subjects, differences emerged with adjustment by genotype. For ESR1-203, levels were significantly lower in tissues homozygous for the main *CC* allele (p = 0.02), and for ESR1-008, levels trended to be higher in bipolar compared schizophrenia subjects in *CT & TT* carriers (p = 0.03) (Figure C in S1 File). Additional trends did not reach significance at p = 0.05.These results suggest effects of disease state and rs2144025 genotype on mRNA expression, but the relationship between ESR1 isoforms appears to be complex.

#### eQTL analysis in a transcriptome database (Brain eQTL Almanac (BRAINEAC))

The BRAINEAC database provides mRNA levels (measured with hybridization arrays) in 10 brain regions of 134 control individuals of European descent, together with genome-wide SNP data. Different probes across the *ESR1* locus enable the analysis of isoforms with selective exons present. Averaged across all 10 regions, rs2144025 was significantly associated with ESR1 mRNA expression for a probe targeting the unique 3' UTR of ESR1-008 (p = 0.009, codominant model). The minor *T* allele was associated with increased expression in heterozygous and homozygous minor samples.

#### eQTL analysis in an RNA-seq database (The Genotype-Tissue Expression (GTEx) project)

The GTEx database[38] provides tissue and transcript-specific RNA-seq data paired with genome-wide SNP data (n = 60–120 samples per brain region at time of analysis). In the nucleus accumbens, the presence of the minor *T* allele of rs2144025 was associated with an increased level of expression of the ESR1-203 transcript (p = 6 x 10^−6^, codominant model). Another association was detected in the amygdala data set (p = 0.045, codominant model), indicating an almost two fold increase in expression levels in carriers of the minor *T* allele. The relatively smaller number of amygdala samples could have accounted for the marginal p value.

#### Association of rs2144025 with brain region size

rs2144025 was found to be associated with amygdala volume in the ENIGMA2 dataset, which compares brain structure volume measurements with genotype data [39]. The presence of the minor allele of rs2144025 was associated with a moderate decrease in amygdala volume (p = 0.009). A subsequent study by the ENIGMA Schizophrenia working group comparing subcortical brain volume in schizophrenia case-control sets noted that amygdala and nucleus accumbens volume was decreased in schizophrenia cases, but that males experienced a smaller deficit than females, suggesting a sex-specific effect in the disease state [40].

#### Effect of rs2144025 on RNA folding

Recent study has identified three new exons inside intron 4 region of ESR1 [41]. Variants containing these exons encode C-terminal truncated ESR1 isoforms, which showed constitutive activity in the absence of estrogen (same reference as above).These isoforms are all expressed in the brain (data not shown). In addition, two more exons were identified by RNA-seq (GTEx). rs2144025 is ~2000 away from one of the exons, potentially affecting the splicing of these exons. Moreover, intronic SNP can affect nascent RNA elongation via changing RNA folding.Single stranded nucleic acid folding analysis of 650 base pairs surrounding rs2144025 (*T>C*) using Mfold software [42] revealed differences between the free energies (ΔG) of the two alleles in folding structure (ΔΔG = -3.9) which could affect regulatory factor binding or pre-mRNA processing (Figure D in S1 File). The *C* allele is more likely to be single stranded (12 out of 23 folding calculations) than the *T* allele (7 out of 21), which may suggest another mechanism for functional differences in expression of ESR1-008.

Taken together, these results support the notion that*ESR1* rs2144025 is a regulatory variant in brain tissues, prompting GWAS analyses of mental or behavioral disorders or traits. Because we test a single candidate variant, rs2144025, a genome-wide corrected p value threshold is not applicable. To test further whether any significant associations of rs2144025 with clinical traits are unique or shared with other *ESR1* SNPs, we subsequently extended the association analysis to numerous other GWAS SNPs (>100) embedded in the large *ESR1* locus.

### Clinical association studies with rs2144025

As rs2144035 has yet to be noted in GWAS studies and ESR1 variants have been associated with behavioral abnormalities, our analysis focused on association with psychological traits, either individually or as a group, such as in psychiatric diagnoses. The trait documentation varied between accessed GWAS cohorts, and only a limited number of traits were typically suitable for analysis, depending on completeness of the records.

#### Age and sex related associations of ESR1 rs2144025 in the bipolar disorder GAIN study

**Fig 2:**
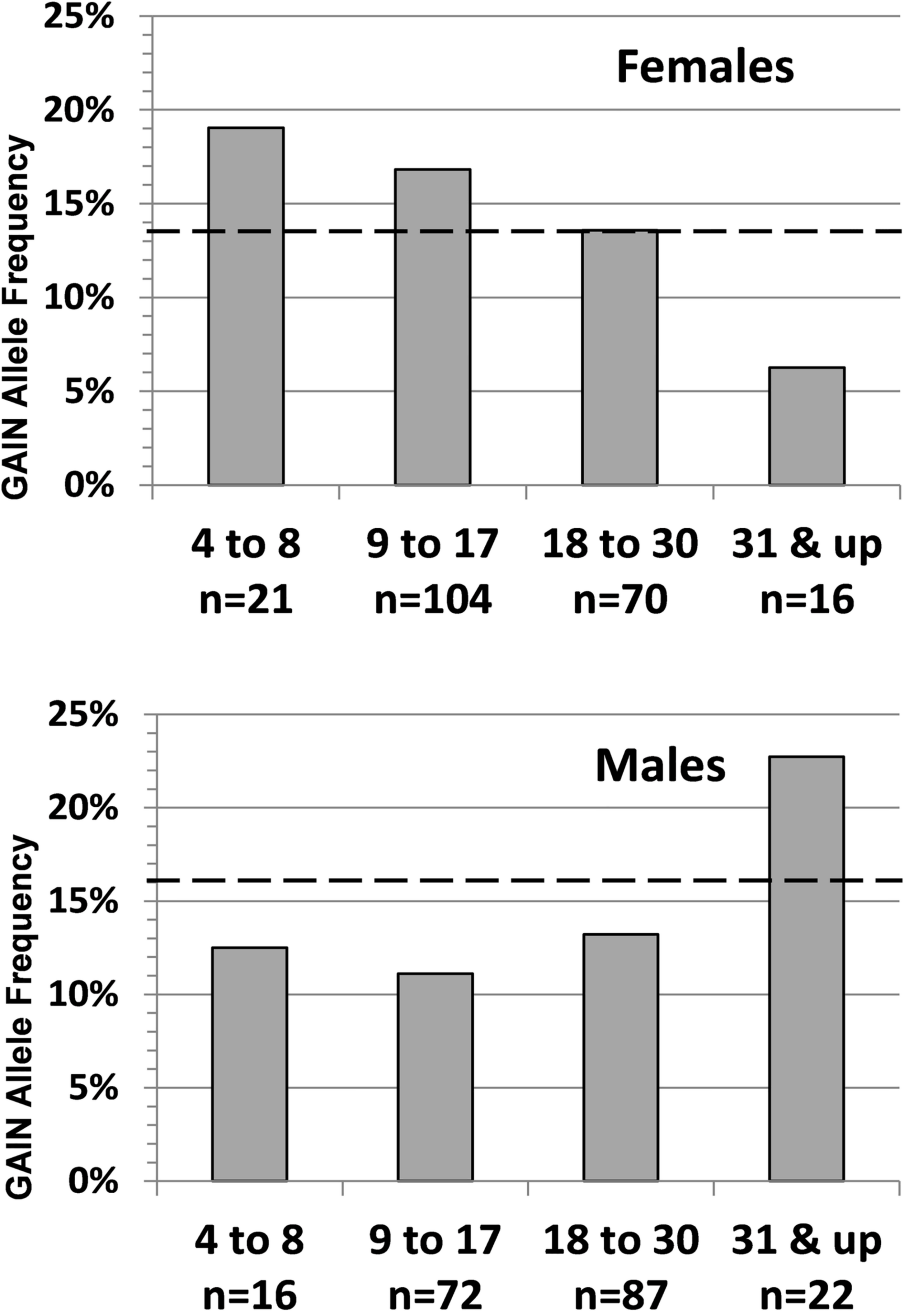
**Minor allele frequency (MAF) of rs2144025 and age of onset in 211 females (A) and 197 males (B) with bipolar disorder in GAIN** (dotted lines: MAF in GAIN controls; f = 12.8%; males = 15.6%). Age of BD onset was nominally associated with rs2144025 in females (additive model p = 0.04), but did not reach significance in males (p = 0.29).

Association of rs2144025 with BD was tested in GAIN, with 512 male and 509 female bipolar subjects and 532 male and 504 female controls of European descent. Examining males and females separately, a case-control analysis indicated that rs2144025 was nominally significant for the presence of bipolar disorder in females in a recessive model (p = 0.03) (Tables 3 & 4), but not in males. There were no violations of HWE in cases or controls from either group. Moreover, rs2144025 was associated with age-of-onset in a subset of 211 females (p = 0.04 in an additive model). MAF was higher in the patients with younger age-of-onset and decreased steadily with age (Fig 2A) from 19 to 6.5% in the older patients. In contrast, in males the MAF increased in the oldest age-of-onset group, but this result did not reach formal significance (Fig 2B).Since detecting robust association with BD in a case-control design would require larger cohorts, we focus on psychological symptoms, affected by *ESR1* variants.

**Table 3:** Associations of rs2144025*C>T* with bipolar bisorder in the GAIN Bipolar Disorder study.

GAIN Bipolar Disorder & Controls	MAF Case %	MAF Con. %	Allelic Test Chi-Squared P	Odds Ratio (95%CI)	Recessive Test Chi-Squared P	Recessive Odds Ratio (95%CI)
Females n = 1013	15.6	12.9	0.08	1.25 (0.97–1.60)	0.03	3.0 (1.09–8.40)
Males n = 1044	13.3	15.6	0.13	0.83 (0.65–1.05)	0.26	0.63 (0.29–1.1)

Case-control analysis of rs2144025 with affected subjects *versus* controls (males: 512 cases and 532 controls; females: 509 cases and 504 controls).

**Table 4:** Associations of rs2144025*C>T* with bipolar episode subtype in the GAIN Bipolar Disorder study.

GAIN BD Type of Episodes	MAF %	Females	
		Corr.P	Corr.R
Depressive	15.1	0.56	-0.03
Manic	14.9	0.59	-0.03
Hypomanic	16.0	0.0006	0.18

Allelic association tests of rs2144025 in GAIN with number of depressive, manic or hypomanic episodes in 181 bipolar females (MAF = 0.16; 51 *CT* and 3 *TT* carriers)

Behavioral phenotype data on BD symptoms were available in 180 females and 158 males, including number of mania, hypomania, and depressive episodes. Testing these phenotypes with rs2144025 revealed an association with number of hypomania episodes in females but not males (p = 0.0006, Tables 5 & 6; additive model p = 0.0004; uncorrected for number of phenotypes (n = 3) considered). The minor *T* allele is associated with a higher number of episodes than the *C* allele (T test p = 0.007).

**Table 5:** Association of rs2144025 with grandiose delusions in both male and female Caucasian schizophrenia patients from the GAIN Schizophrenia GWAS.

GAIN Schizophrenia Grandiose delusions	Chi-Squared P	Fisher's Exact P	Regression Odds Ratio	OR Confidence limits
Allelic test	0.002	0.002	0.67	0.52–0.86
Dominant allele test	0.0004	0.0005	0.60	0.45–0.80
Recessive allele test	0.99	1	0.99	0.44–2.23

**Table 6:** Subject count and allele frequency information for the grandiose delusions diagnosis in Caucasian schizophrenia patients from the GAIN Schizophrenia GWAS.

Grandiose Delusions	n	MAF	Sex	n
Yes	511	12%	Male	706
No	507	17%	Female	312

The lower panel contains subject count and allele frequency information.

#### Associations of ESR1 rs2144025 in the GAIN Schizophrenia study

In this GWAS study, rs2144025 is significantly associated with several behavioral traits but not schizophrenia *per se*. The most significant association in this Caucasian population of schizophrenic subjects was observed with grandiose delusions when testing both males and females together (dominant allele test p = 0.0004; Table 4). An odds ratio of 0.67 suggested a protective effect of the minor *T allele*. Of the 1018 Caucasian subjects with information on grand delusions, 706 were males and 312 females, with approximately half experiencing grand delusions. Additional behavioral phenotypes were also available in the data set, including delusionary and hallucinatory traits. The pattern of positive and negative symptoms was also significant (p = 0.003), while alogia was weakly associated (p = 0.03).

#### Association of ESR1 rs2144025 in the International Multi-Center ADHD Genetics Project

While rs2144025 was not significantly associated with ADHD, we discovered associations with comorbid psychiatric symptoms. The cohort contains significantly more males than females, reflecting the strong ADHD sex difference in the general population. Considering all subjects, rs2144025 is associated with the presence of psychiatric symptoms (p = 0.005 in a dominant allele test; OR = 1.5), but when the subjects are stratified by age of assessment, the association with rs2144025 strengthens (dominant allele test p = 2.4e-5; OR = 2.1) in the group age 10 or older (Table 7). This association is significant in both males only (p = 0.0003; OR = 2.5, dominant test) and females at a p value of 0.04 (OR = 3.4, dominant test), the less significant level reflecting the low number of female subjects in that age group (n = 58).

**Table 7:** Associations of rs2144025 with comorbid psychiatric diagnoses in the GAIN Attention Deficit Hyperactivity Disorder study.

GAINADHD(n)	Psych.Dx(n)	MAF Cases (%)	MAF Control (%)	Chi-SquaredP	Odds Ratio (95% CI)	OR Confidence limits	Dominant Chi-Squared P	Dominant Odds Ratio	Dominant OR Confidence limits
All subjects over 10 n = 485	100	21.5	11.6	0.0003	2.1	1.4–3.1	2.4E-05	2.7	1.7–4.3
Males over 10 n = 427	82	20.7	11.6	0.002	2.0	1.3–3.1	0.0003	2.5	1.5–4.2
Females over 10 n = 58	18	25.0	11.3	0.06	2.6	0.9–7.3	0.04	3.4	1.1–11.3

Allelic and dominant (3 columns on the right) association tests were performed in all ADHD subjects and in boys and girls of age 10 or more (cases with psychiatric diagnoses and controls without diagnoses).

#### Associations of ESR1 rs2144025 in the eMERGE study from the Children’s Hospital Boston

In this population study of children, rs2144025 is associated with psychological diagnoses in Caucasian girls, but not boys, in dominant and allelic tests (p = 0.002 and p = 0.01, respectively, OR = 3.2, Table 8). Psychological diagnosis in this GWAS is defined as ICD9 codes 296–313, which include episodic mood disorders such as BD, mood and anxiety disorders but not autism, schizophrenia, or ADHD. Upon consideration of the hormonal surge that occurs at the onset of puberty and the possible effect it may have on ESR1 signaling, an analysis of females over the age of 9 demonstrated a strengthened association (dominant allele test p = 0.0009; OR = 4.7), while no significant association was found in females under the age of nine.

**Table 8:** Associations of rs2144025 in eMERGE GWAS with psychological disorders of Caucasian children (eMERGE Boston Children’s Hospital).

eMERGE	MAF %	Dx(n)	Allelic Test Chi-Squared P	Odds Ratio (Minor Allele)	Dominant Chi-Squared P	Odds Ratio (95%CI)	Average Age (Years)
Females n = 278	13.7	31	0.01	2.26(1.19–4.30)	0.002	3.20 (1.49–6.87)	9.2±5.2
Females			0.006	2.74(1.30–5.76)	0.0009	4.68(1.78–12.3)	13.2±3.1
9 and over n = 149	16.4	21					
Females			1	0.95(0.21–4.32)	0.96	1.04(0.21–5.25)	4.4±2.3
8 and under n = 129	10.5	10					
Males n = 449	13.3	74	0.53	1.18(0.71–1.94)	0.55	1.18(0.68–2.07)	8.7±4.9

Psychological diagnosis is defined as ICD9 codes 296–313. Anxiety and episodic mood disorders such as bipolar disorder were included. Average age, females 9.2±5.0 years, range 1–21, n = 278; females with psychological diagnosis: 11.0±4.8 years, n = 31; males: average age 8.7±4.9 years, range 1–21; males with psychological diagnosis: 10.2 ±.3 years, n = 74.

#### Testing additional ESR1 SNPs available in the four GWAS for comparison to rs2144025

While thus far we have treated the association analysis as a single SNP candidate hypothesis with rs214402, we also analyzed all other available SNPs in the *ESR1* gene locus. In the GAIN BD GWAS with 104 SNPs in *ESR1*, rs2144025 remains the most significant SNP associated with number of hypomania episodes in females (Table E in S1 File). In males, other SNPs are also associated(Table E in S1 File); however, the MAF of each significant SNP in males is <2%, raising concern about cohort size and population stratification, while rs2144025 has a minor allele frequency of ~0.15.These results further support a role of rs2144025 specifically in modulating a trait in bipolar disorder. Similar results were obtained in GAIN schizophrenia (Table F in S1 File) and in the ADHD cohort study (Table G in S1 File). In the eMERGE study from Children’s Hospital Boston with 107 *ESR1* SNPs (Table H in S1 File), several SNPs display a slightly stronger association than rs2144025, but these SNPs tend to be in LD with each other and reside on the opposite allele to rs2144025.Upon splitting all of the psychiatric disorders into three subgroups–Anxiety, Depression, and Other–it appears that the apparent strength of the association in these other SNPs is primarily driven by the subset of individuals with anxiety. Possibly, the haplotype harbors another functional variant; however, these SNPs did not replicate in the other GWAS cohorts analyzed.

## Discussion

We identify here a common *ESR1* SNP, rs2144025-*C>T*, as a possible regulatory variant affecting ESR1 expression, and behavioral traits in mental diseases. Assignment of rs2144025 as a regulatory variant was supported by a significant association with allelic mRNA expression observed in PFCs of schizophrenic and bipolar subjects, suggesting that the effects of rs2144025 be amplified in the disease state. Similar changes under pathophysiology had been observed for *DRD2* SNPs that alter splicing [43], in opposite directions in normal subjects and schizophrenics [44], and ESR1 isoform expression had been shown to change in autopsy brain tissues from schizophrenics [12]. Supporting a role in behavioral modification, rs2144025 is associated with various psychological traits in four GWAS, in a sex and age dependent fashion in some but not all GWAS. Possibly, ESR1-008 mRNA isoforms could act in both estrogen dependent and independent modes, with varying effects on males and females at various ages, such as post-puberty.

### Genomic features of rs2144025

Sequencing and LD analysis of the ESR1 locus failed to reveal other variants that could account for the large allelic mRNA expression imbalance in the Stanley PFC samples. Located in intron4 and proximity to several recently identified exons, rs2144025 may act by altering RNA folding dynamics (Figure C in S1 File), thereby affecting pre-mRNA processing. Large allelic mRNA ratios in the diseased PFCs were detectable only with a marker SNP in exon4 (Fig 1); this exon is present in the ESR1 mRNA isoform ESR1-008, which is the main isoform in the brain according to GTEx data. In contrast, the other main brain isoform identified in GTEx, ESR1-203, detected here selectively with a 3’UTR marker SNP in an exon lacking in ESR1-008, did not display large allelic ratios in PFC samples. Both isoforms were readily detectable with qRT-PCR in brain. Lacking some 5’ and 3’UTR regions, and a portion of the ligand binding domain, ESR1-008 is considered to signal independently of a ligand but appears also to respond to estrogens, can heterodimerize, and might act as a transcriptional repressor or enhancer (depending on the gene locus) [1, 2].As a further support of our conclusions, we found that rs2144025 is detected as a *cis-*eQTL for ESR1 RNA expression in brain tissues (from BRAINEAC data), primarily within probes directed against the unique 3'UTR associated with ESR1-008, and for ESR1-203 within specific brain regions (from GTEx data). Other isoforms are also detectable in brain tissues [12], and the relationships of rs2144025 with formation of ESR1 mRNA isoforms remains to be determined. Owing to the complexity of the ESR1 locus, a definitive assessment of a functional mechanism cannot be assigned to rs2144025.

### Clinical phenotype associations of rs2144025

Multiple *ESR1* variants had been implicated in diverse phenotypes, but causative effects have remained unknown, except for acquired mutations in cancer. In candidate gene association studies, rs2144025 had been implicated in one study related to breast cancer (Table A in S1 File and references therein). Candidate gene studies have further identified two *ESR1* SNPs showing similar allele frequency and LD D’>0.5, associated with temperament and suicidal attempts (rs974276) and as a risk factor in schizophrenia (rs2273206) (Tables A & B in S1 File). In addition, numerous *ESR1* SNPs have been implicated in a variety of clinical phenotypes by GWAS (Table B in S1 File). Many of these SNPs are in high LD (D’) with rs2144025 but with low MAF; therefore, these variants may not represent clinical associations involving rs2144025, which has high minor allele frequency across populations (12–33%). Additional GWAS-implicated SNPs showing intermediate LD (D’>0.5; extracted from SNAP[45]) with rs2144025 (Table C in S1 File) include rs2179922 and rs932477 associated with cognitive decline [20]. The vast majority of these variants are likely to be only surrogate markers for rs2144025 and/or additional regulatory variants yet to be discovered.

Pursuing the observation of large effects of rs2144025 on allelic mRNA expression in bipolar and schizophrenic brain tissues, we queried four separate GWAS for associations of behavioral phenotypes with rs2144025.The GWAS encompass children and adults, and were analyzed with age and sex as co-variates, recognizing the importance of hormonal differences. In the GAIN GWAS of bipolar disorder, a significant association was observed with hypomanic episodes, again only in females (p = 0.0006). Hypomania, an uncharacteristic persistent euphoric, expansive or irritable mood, is less severe than mania and a feature of bipolar II disorder. Taken together, the results suggest that rs2144025 modulates behavioral traits in BD females. Future studies also have to account for possible influence of rs2144025 on age of onset of BD in males and females.

In the GAIN schizophrenia GWAS, rs2144025 again reveals associations with behavioral symptoms, for example ‘grandiose delusions’ (p = 0.0004).This result further supports the*ESR1* variant function as a disease modifier, affecting behavioral symptoms. Extending our study to the International Multi-Center ADHD Genetics Project, a disorder affecting boys disproportionally more than girls, we find rs2144025 associated with comorbid psychiatric symptoms (p = 0.00002) in both boys and girls with age ≥10.

In view of the apparent dependence of rs2144025 on age and sex, we turned to a Caucasian pediatric cohort in eMERGE, revealing an association of rs2144025 with diagnoses of psychological disorders. This association was observed primarily in girls age 9 years and above (p = 0.0009); the lack of any effects in younger girls reinforces the conclusion that estrogen hormonal activity is an important variable determining the impact of rs2144025, which may modify multiple psychological phenotypes as a function of hormonal status. Similarly, earlier studies had identified the involvement of *ESR1* SNPs in psychological phenotypes specifically in females [5, 15, 17, 46]. Thus, *ESR1* variants could have broad impact on sexually dimorphic diseases, with rs2144025 affecting behavioral traits in females, and also in males–the latter less well studied–depending on the conditions. The results presented here guide further studies by emphasizing potential interactions between regulatory *ESR1* variants, disease status, sex, and age.

The association studies described above are based on a single candidate SNP hypothesis, namely, rs2144025 is a regulatory variant with influence on psychiatric traits. Even when adjusting for the analysis of multiple traits, rs2144025 remains significant in each study cohort. Because of the large size of the *ESR1* gene locus and the numerous SNPs already implicated in various disorders, only some of which are in partial LD with rs2144025, we asked whether rs2144025 stands out among all other SNPs present in the *ESR1* gene locus, compared to the p values achieved with rs2144025 (Tables E-I in S1 File). Indeed, rs2144025 scored either best or near the top among the >100 SNPs available for analysis. This result strongly supports a role of rs2144025 in behavioral traits. Previous research had suggested that efficacy of estrogen-based therapies may be contingent upon genotype status of *ESR1*[12], an issue requiring further analysis to assess treatment outcomes.

#### Study limitations

As clinical genetic associations taken alone are prone to false positive results, further replications of the conditional effects of rs2144025 are needed. The pervasive literature on the role of *ESR1* variants in multiple clinical studies and diseases supports the findings in this study, but causative relationships had remained elusive. Our molecular genetics results identifying rs2144025 as a candidate variant, alleviating the need for multiple hypotheses corrections. The molecular mechanisms underlying the effects of rs2144025 require more study, challenged by the location in the large 67 kb intron4 and the highly complex genomic stricture of the *ESR1* gene locus. We further caution that assignment of ESR1 RNA isoforms on the basis of RNAseq data in GTEx may not reflect all isoforms present or include short sequence reads assigned to a false isoforms; however, this caveat does not affect the associations observed with rs2144025 in clinical studies.

## Materials and methods

### Human tissue samples

We obtained genomic DNA and total mRNA from Brodmann’s area 46 (dorsolateral prefrontal cortex, termed here PFC) in 105 individuals from the Stanley Medical Research Institute. The 28s/18s absorbance ratios were 2.13 +/- 0.59, indicating sufficient RNA integrity. The cohort included 35 controls (females n = 9), 35 subjects with bipolar disorder (females n = 18), and 35 subjects with schizophrenia (females n = 9). The Stanley brain collection specimens were collected by participating medical examiners between January 1995 and June 2002, with informed consent from next-of-kin. All subjects were of European descent, and median age at death was 44±9 years. Average post-mortem interval was 32.9±16.0 hours.RNA integrity and purity were determined with an Agilent 2100 Bioanalyzer by the Stanley Medical Research Institute. There was no correlation between AEI ratios and [28s]/[18s] RNA ratios or PMI. For this study, the samples were unblinded with respect to diagnosis. The second cohort consisted of 214 subjects from the Miami Dade Brain Bank and included 119 subjects who were cocaine addicts (females n = 18) and 95 controls (females n = 13). Approximately 35% were of African descent, 24% were Hispanic Caucasian, and 41% were of European descent. The median age of death was 35 ± 10. Prefrontal cortex and putamen tissues were available for this group. This study of deidentified autopsy tissues was judged not to qualify as human experimentation, and the need for IRB approval was waived for this study by the OSU IRB committee.

### cDNA synthesis

Reaction conditions had been described previously [47]. Oligonucleotide primers are listed below. Following reverse transcription (RT), 1 μl cDNA was preamplified with 0.5 μl 0.1 μM gene-specific forward and reverse primers covering regions of interest, including β-actin, in 30 μl PCR mix for six 60 minute extension cycles to enhance specificity of the reactions.

Actin F 5’CCTGGCACCCAGCACAAT; Actin R 5’GCCGATCCACACGGAGTACT
rs3798577 F 5’TGGTGTTGCATTTAGCCCTGG; rs3798577 R 5’AGCCACAACAATCCTGCACA (3’UTR of ESR1-203)
rs1801132 F 5’CAGTGCCTTGTTGGATGCTG; rs1801132 R 5’CCCTGTCTGCCAGGTTGGT (exon4 of ESR1-008)
F: forward primer; R: reverse primer.

### Quantitative mRNA analysis by RT-PCR

The procedure for RT-PCR has been described [47]. *ESR1* and β-actin transcripts were amplified with the primers listed above.

### SNaPshot procedure to measure allelic mRNA ratios

This primer extension method [47, 48] was applied to samples heterozygous for two marker SNPs located in *ESR1* exons: rs3798577 and rs1801132. cDNA was made from total RNA with both poly-*dT* and the reverse primers listed above to minimize any effects of partial degradation of the mRNA. A 70bp fragment incorporating the marker SNP was PCR amplified in cDNA (equivalent to ~10 ng total RNA per assay) and genomic gDNA, and allelic ratios measured with SNaPshot, with results normalized to gDNA as described [48]. Each cDNA sample was measured independently at least three times. PCR primers were identical to those listed above, while extension primers were:

3’UTR rs3798577 PEP 5’GGCATGGAGCTGAACAGTAC
Exon4 rs1801132 PER 5’GTAGGATCATACTCGGAATAGAGTAT

Average cDNA ratios had a S.D. of ±0.12 for rs3798577 and ±0.13 for rs1801132, while gDNA ratios had SD ±0.06 and ±0.05, respectively.

### Genotyping assays

Standard genotyping methods were employed to determine selected SNP genotypes [14]. For the *TA* VNTR (rs71685044), the repeat number ranged from 10 to 30 repeats. When amplified with PCR, the fragment size varied between 160 to 200 bp with two peaks centered around 172 and 190. As previously reported[14, 49], we treated the *TA* repeat as a biallelic variant, with a short form (fragment length 178 bp and lower) and a long form (fragment length 180 bp and higher).

### Sequencing of the genomic *ESR1* locus

Targeted regions of interest (1–5 kb) were amplified by PCR from genomic DNA of 9 Stanley PFC samples. Library preparation and PGM ion torrent sequencing has been described elsewhere [50]. Reads were analyzed with CLC Bio software.

### GWAS data sets

Access to GWAS data was supported by a local umbrella IRB protocol (OSUMC), with specific approval for each GWAS set by the OSUMC review committee.

1. Genetic Association Information Network (GAIN) Whole Genome Association of Bipolar Disorder version 3 (Accession: phs000017.v3.p1).
  phs000017.v3.p1.c2 (BARD) bipolar and related disorders, 841 subjects
  phs000017.v3.p1.c1 (GRU) general research use, 1767 subjects
  phs000017.v3.p1.c3 (BDO) bipolar disorder only, 653 subjects
2. Genetic Association Information Network (GAIN) Genome-Wide Association Study of Schizophrenia version 3 (Accession: phs000021.v3.p2).
  phs000021.v3.p2.c1 (GRU) general research use, 4589 subjects
  phs000021.v3.p2.c2 (SARC) schizophrenia and related conditions, 475 subjects
3. International Multi-Center ADHD Genetics Project (Accession: phs000016.v2.p2), part of Genetic Association Information Network (GAIN).
  phs000016.ADHD.v2.p2.c1, ADHD and its complications, 2758 subjects
4. The Gene Partnership (TGP)—eMERGE Data Distribution Set 1 (Accession:phs000495.v1.p1), part of the National Human Genome Research Institute.
  phs00495.eMERGE_Pediatric_CHB.v1.p1.c1.HR_CHB: 1024 subjects, for the study of genetic and environmental factors that contribute to childhood health, development, and disease.

### Statistical analysis

#### Association ofESR1 variants withallelic mRNA ratios and with mRNA levels

An allelic expression imbalance (AEI) was defined as an allelic mRNA ratio (after normalization to the ratio in gDNA) equal to or greater than 1.4. As AEI ratios varied over a wide range, and the large *ESR1* locus is likely to harbor more than one regulatory variant, we divided RNA samples into those with no significant AEI, moderate AEI (ratio 1.4 to 1.9) and large (2 and above). To assess associations between *ESR1* variants and AEI categories, *ESR1* genotypes were converted to 0_1 for heterozygous and 0_0 or 1_1 for homozygous for either allele. Variants in *ESR1* were then screened for association with AEI status (no AEI *versus* moderate AEI or large AEI), using F test statistics and Bonferroni correction for number of SNPs tested. Across samples for which AEI was obtained, the 28s/18s absorbance ratio was 2.18 +/- 0.57, which was not significantly associated with measured AEI (p > 0.05). Gene expression data were analyzed utilizing the R-package SNPassoc[51].

#### Association of *ESR1* variants with clinical traits

Genetic data were analyzed with genetic analysis software SVS (Golden Helix, Inc.). Covariates in all analyses included sex and age. Trait selection focused on behavioral traits either individually or combined under psychological diagnosis. The following phenotypes were utilized: case/control bipolar disorder (presence of any psychological diagnosis, age-of-onset and number of hypomanic, manic, and depressive episodes in bipolar disorder), schizophrenia (alogia, grandiose delusions), ADHD (co-morbid psychological disorders), and The Gene Partnership (TGP) population study (any psychological disorders) (ICD9 codes 296–313). Number of eligible subjects with specific traits recorded varied between cohorts. P values were reported without Bonferoni correction.

## Supporting information

S1 File Supporting information file. This file includes all supporting figures and tables cited within the text.
(DOCX)
