# Supplementary material for: Intronic SNP in ESR1 encoding human estrogen receptor alpha is associated with brain ESR1 mRNA isoform expression and behavioral traits
Source: PLoS One. 2017 Jun 15;12(6):e0179020. doi: 10.1371/journal.pone.0179020 (PMC5472281; doi:10.1371/journal.pone.0179020)
Supplement: S1 File — This file includes all supporting figures and tables cited within the text. (DOCX) [file pone.0179020.s001.docx]

Frequent intronic SNP in human estrogen receptor *ESR1* influences brain mRNA expression and behavioral disorders

Julia K. Pinsonneault, John T. Frater, Benjamin Kompa, Roshan Mascarenhas, Danxin Wang and Wolfgang Sadee

**Supplemental Data**

| **Location** | **position** | **dbSNP rs#** | **MAF** | **Bone/Joint** | **Cancer** | **Cardio** | **CNS** | **Infection** | **Fertility** | **References** |
| --- | --- | --- | --- | --- | --- | --- | --- | --- | --- | --- |
| 5' near gene | 152009638 | rs2941740 | 0.34 | X |  |  |  |  |  | ([1](#_ENREF_1)) |
| upstream Intron | 152089768 | rs2485209 | 0.41 | X |  |  |  |  |  | ([2](#_ENREF_2)) |
| upstream Intron | 152119119 | rs2881766 | 0.41 |  |  | X |  |  |  | ([3](#_ENREF_3)) |
| upstream Intron | 152121442 | rs11964281 | 0.05 |  |  | X |  |  |  | ([4](#_ENREF_4)) |
| upstream Intron | 152125231 | rs34535804 | 0.19 |  |  |  | X |  |  | ([5](#_ENREF_5)) |
| upstream Intron | 152127664 | TA repeat | 0.43 |  |  |  | X |  |  | ([6](#_ENREF_6)) |
| Exon1 | 152129077 | rs2077647 | 0.43 | X | X |  | X | X |  | ([7-11](#_ENREF_7)) |
| Exon1 | 152129308 | rs746432 | 0.05 |  |  |  | X |  |  | ([10](#_ENREF_10)) |
| Intron1 | 152130918 | rs532010 | 0.38 |  |  |  | X |  |  | ([10](#_ENREF_10)) |
| Intron1 | 152140042 | rs3844508 | 0.27 |  |  |  | X |  |  | ([12](#_ENREF_12)) |
| Intron1 | 152161066 | rs9397448 | 0.41 | X |  |  |  |  |  | ([13](#_ENREF_13)) |
| Intron1 | 152162317 | rs9322331 | 0.17 |  |  | X |  |  |  | ([14](#_ENREF_14)) |
| Intron1 | 152163334 | rs8179176 | 0.45 |  |  |  | X |  |  | ([15](#_ENREF_15)) |
| Intron1 | 152163335 | rs2234693 | 0.44 | X | X | X | X | X |  | ([7](#_ENREF_7), [8](#_ENREF_8), [11](#_ENREF_11), [13](#_ENREF_13), [16-27](#_ENREF_16)) |
| Intron1 | 152163381 | rs9340799 | 0.26 | X | X | X | X | X |  | ([7](#_ENREF_7), [14-17](#_ENREF_14), [20-27](#_ENREF_20)) |
| Intron2 | 152200430 | rs9322336 | 0.23 |  | X |  |  |  |  | ([28](#_ENREF_28)) |
| Intron2 | 152201624 | rs9340844 | 0.03 |  |  | X |  |  |  | ([29](#_ENREF_29)) |
| Intron3 | 152203104 | rs6557170 | 0.26 |  |  |  | X |  |  | ([30](#_ENREF_30)) |
| Intron3 | 152208722 | rs1913474 | 0.28 |  |  | X |  |  |  | ([31](#_ENREF_31)) |
| Intron3 | 152229850 | rs2347867 | 0.44 |  |  | X |  |  |  | ([32](#_ENREF_32)) |
| Intron3 | 152236879 | rs9397453 | 0.04 |  |  | X |  |  |  | ([29](#_ENREF_29)) |
| Intron3 | 152241150 | rs988328 | 0.22 |  |  | X |  |  |  | ([3](#_ENREF_3)) |
| Intron3 | 152247152 | rs9397456 | 0.26 |  |  | X |  |  |  | ([33](#_ENREF_33)) |
| Intron3 | 152260642 | rs9371562 | 0.03 |  |  | X |  |  |  | ([29](#_ENREF_29)) |
| Exon4 | 152265522 | rs1801132 | 0.26 | X |  | X |  | X |  | ([7](#_ENREF_7), [31](#_ENREF_31), [34](#_ENREF_34)) |
| Intron4 | 152265659 | rs9397459 | 0.04 |  |  | X |  |  |  | ([29](#_ENREF_29)) |
| Intron4 | 152270672 | rs3020314 | 0.43 | X | X | X |  |  |  | ([33-35](#_ENREF_33)) |
| Intron4 | 152278741 | rs3020317 | 0.29 |  |  | X |  |  |  | ([31](#_ENREF_31), [33](#_ENREF_33)) |
| Intron4 | 152283279 | rs1884051 | 0.43 | X |  |  |  |  |  | ([34](#_ENREF_34)) |
| Intron4 | 152285687 | rs2982694 | 0.18 |  |  | X |  |  |  | ([36](#_ENREF_36)) |
| Intron4 | 152286625 | *rs985694 | 0.25 |  |  | X |  |  |  | ([31](#_ENREF_31), [33](#_ENREF_33)) |
| Intron4 | 152291366 | rs1884052 | 0.18 | X |  |  |  |  |  | ([37](#_ENREF_37)) |
| Intron4 | 152295613 | rs9383951 | 0.07 |  |  | X |  |  |  | ([29](#_ENREF_29)) |
| Intron4 | 152297100 | *rs2179922 | 0.15 | X |  |  |  |  |  | ([38](#_ENREF_38)) |
| Intron4 | 152302578 | rs726281 | 0.50 |  |  |  | X |  |  | ([39](#_ENREF_39)) |
| Intron4 | 152303437 | rs728524 | 0.12 |  |  |  | X |  |  | ([15](#_ENREF_15)) |
| Intron4 | 152304596 | *rs932477 | 0.20 |  |  | X |  |  |  | ([31](#_ENREF_31)) |
| **Intron4** | **152307706** | **rs2144025** | **0.33** |  | **X** |  |  |  |  | **(**[**40**](#_ENREF_40)**)** |
| Intron4 | 152317140 | rs7757956 | 0.14 |  |  | X |  |  |  | ([31](#_ENREF_31)) |
| Intron4 | 152322885 | rs722208 | 0.41 |  | X |  |  |  |  | ([41](#_ENREF_41)) |
| Intron4 | 152323192 | rs722207 | 0.38 |  |  |  | X |  |  | ([42](#_ENREF_42)) |
| Intron4 | 152326197 | rs6905370 | 0.41 |  | X |  |  |  |  | ([43](#_ENREF_43)) |
| Intron4 | 152328616 | rs1569788 | 0.41 |  |  | X |  |  | X | ([4](#_ENREF_4), [44](#_ENREF_44)) |
| Intron4 | 152330673 | rs9340958 | 0.07 |  |  |  |  |  | X | ([45](#_ENREF_45)) |
| Intron5 | 152333945 | rs9340978 | 0.05 |  |  |  |  |  | X | ([45](#_ENREF_45)) |
| Intron5 | 152371190 | rs3020368 | 0.09 |  |  | X |  |  |  | ([31](#_ENREF_31)) |
| Intron5 | 152376524 | *rs6932902 | 0.23 |  |  |  |  |  | X | ([46](#_ENREF_46)) |
| Intron5 | 152380515 | *rs9397080 | 0.24 |  | X |  |  |  |  | ([47](#_ENREF_47)) |
| Intron6 | 152382311 | *rs2273206 | 0.24 |  |  |  | X |  |  | ([48](#_ENREF_48)) |
| Intron6 | 152382325 | rs2273207 | 0.15 |  |  |  | X |  |  | ([48](#_ENREF_48)) |
| Intron6 | 152382382 | rs2207396 | 0.23 |  |  | X |  | X | X | ([7](#_ENREF_7), [45](#_ENREF_45), [49](#_ENREF_49)) |
| Intron6 | 152382420 | *rs974276 | 0.24 |  |  |  | X |  |  | ([42](#_ENREF_42)) |
| Intron6 | 152389968 | rs3020375 | 0.49 |  |  |  |  |  | X | ([50](#_ENREF_50)) |
| Intron6 | 152396036 | rs7766585 | 0.23 |  | X |  |  |  |  | ([51](#_ENREF_51)) |
| Intron7 | 152418575 | rs3778099 | 0.19 | X |  |  |  |  |  | ([37](#_ENREF_37)) |
| Exon8 | 152420095 | rs2228480 | 0.18 |  |  | X |  |  |  | ([52](#_ENREF_52)) |
| 3'UTR | 152421130 | rs3798577 | 0.44 |  |  |  | X |  |  | ([39](#_ENREF_39), [53](#_ENREF_53)) |
| 3'UTR | 152421854 | rs3798758 | 0.13 |  |  | X |  |  |  | ([52](#_ENREF_52)) |
| 3'UTR | 152422335 | rs2747648 | 0.01 |  |  |  | X |  |  | ([54](#_ENREF_54)) |
| 3'UTR | 152423905 | rs1062577 | 0.14 |  | X |  |  |  |  | ([55](#_ENREF_55)) |

**Table A**: *ESR1* variants identified in candidate associations studies of various clinical phenotypes. Phenotypes were grouped into six categories: Bone/Joint, Cancer, Cardio, CNS, Infection, and Fertility. Bone/Joint includes but is not limited to bone density, body height, osteoarthritis, and rheumatoid arthritis. Cardio includes but is not limited to blood pressure, type II diabetes, lipid, and artery phenotypes. Cancer is defined as any cancer or cancer treatment. CNS is defined as any behavioral or developmental brain disorder. “*” defines SNPs with D’>0.5 with rs2144025, with details provided in Supplemental Table 7. Position is location within the reference sequence GRCh37.p10. MAF is defined by the 1000 Genomes project. For variants with multiple associations, not every older citation is included in the table.

| snpID | Position | Phenotype | Pvalue | GWAS PMID |
| --- | --- | --- | --- | --- |
| rs45918598 | 151943639 | Bone mineral density | 4.90E-10 | 19801982 |
| rs10872676 | 151943977 | Bone mineral density | 4.40E-10 | 19801982 |
| rs9383936 | 151944614 | Breast size | 2.20E-09 | 22747683 |
| rs7776340* | 151945666 | Bone mineral density | 4.40E-13 | 22504420 |
| rs12665607 | 151946629 | Breast size | 1.30E-09 | 22747683 |
| rs7751941* | 151946658 | Bone mineral density | 2.00E-24 | 22504420 |
| rs6917575 | 151947539 | Breast size | 1.40E-09 | 22747683 |
| rs6901351 | 151947736 | Breast size | 2.30E-09 | 22747683 |
| rs74295874 | 151947757 | Breast size | 1.20E-09 | 22747683 |
| rs2046211* | 151948284 | Transmission distortion | 5.90E-16 | 22377632 |
| rs2046210* | 151948366 | Breast cancer | 3.60E-39 | 22383897 |
| rs6557161 | 151950235 | Breast size | 9.20E-09 | 22747683 |
| rs7774781 | 151950723 | Breast size | 1.70E-09 | 22747683 |
| rs9397435 | 151951220 | Breast size | 1.10E-09 | 22747683 |
| rs9397436 | 151952002 | Breast size | 8.50E-11 | 22747683 |
| rs9397437 | 151952332 | Breast size | 1.90E-10 | 22747683 |
| rs58343273 | 151953180 | Breast size | 3.40E-10 | 22747683 |
| rs9383590 | 151953765 | Breast size | 3.70E-10 | 22747683 |
| rs9397068 | 151953859 | Breast size | 4.80E-10 | 22747683 |
| rs6900157* | 151954127 | Bone mineral density | 2.20E-09 | 19079262 |
| rs60954078 | 151955914 | Breast size | 2.50E-10 | 22747683 |
| rs9383937 | 151957119 | Breast size | 2.70E-10 | 22747683 |
| rs12173562 | 151957570 | Breast size | 3.50E-10 | 22747683 |
| rs12173570 | 151957714 | Breast size | 5.60E-11 | 22747683 |
| rs6930633 | 151958091 | Bone mineral density | 8.90E-11 | 23074152 |
| rs6912323 | 151958612 | Breast size | 6.00E-10 | 22747683 |
| rs17081533 | 151958815 | Breast size | 5.00E-10 | 22747683 |
| rs852003 | 151962202 | Bone mineral density | 2.60E-15 | 22504420 |
| rs77275268 | 151969198 | Breast size | 6.30E-10 | 22747683 |
| rs9371545 | 151969740 | Breast size | 9.30E-10 | 22747683 |
| rs712219 | 151978439 | Bone mineral density | 7.90E-09 | 18445777 |
| rs3020331* | 152008780 | Bone mineral density | 3.10E-11 | 18445777 |
| rs3020332* | 152008924 | Bone mineral density | 2.80E-09 | 19801982 |
| rs2941741* | 152008982 | Bone mineral density | 2.20E-10 | 19801982 |
| rs2941740* | 152009638 | Bone mineral density | 2.00E-10 | 20096396 |
| rs3020333* | 152010254 | Bone mineral density | 2.90E-10 | 19801982 |
| rs2982573* | 152010534 | Bone mineral density | 2.40E-10 | 19801982 |
| rs2982571* | 152012739 | Bone mineral density | 3.90E-10 | 19801982 |
| rs3020334* | 152012956 | Bone mineral density | 3.50E-10 | 19801982 |
| rs3020335* | 152013223 | Bone mineral density | 4.80E-10 | 19801982 |
| rs2982570* | 152013748 | Bone mineral density | 4.80E-10 | 19801982 |
| rs851982* | 152024985 | Bone mineral density | 7.20E-12 | 18445777 |
| rs2982562 | 152052601 | Bone mineral density | 5.80E-09 | 19801982 |
| rs2982561 | 152052652 | Bone mineral density | 8.90E-09 | 19801982 |
| snpID | Position | Phenotype | Pvalue | GWAS PMID |
| rs3020343 | 152054363 | Bone mineral density | 4.80E-09 | 19801982 |
| rs2982560 | 152055606 | Bone mineral density | 4.30E-09 | 19801982 |
| rs2982558 | 152056146 | Bone mineral density | 7.00E-09 | 19801982 |
| rs2982557 | 152056369 | Bone mineral density | 3.10E-09 | 19801982 |
| rs2982556 | 152056842 | Bone mineral density | 5.00E-09 | 19801982 |
| rs2982554 | 152058010 | Bone mineral density | 4.80E-09 | 19801982 |
| rs3020349 | 152058268 | Bone mineral density | 5.50E-09 | 19801982 |
| rs3020300 | 152058844 | Bone mineral density | 8.00E-09 | 19801982 |
| rs2982552 | 152059563 | Bone mineral density | 1.50E-09 | 19801982 |
| rs2982551 | 152061210 | Bone mineral density | 2.10E-09 | 19801982 |
| rs3020301 | 152061579 | Bone mineral density | 2.00E-09 | 19801982 |
| rs1415194 | 152063998 | Bone mineral density | 5.00E-10 | 19801982 |
| rs1999807 | 152064199 | Bone mineral density | 5.20E-10 | 19801982 |
| rs3020304 | 152064464 | Bone mineral density | 1.70E-09 | 19801982 |
| rs3020306 | 152065886 | Bone mineral density | 4.80E-10 | 19801982 |
| rs3020307 | 152067244 | Bone mineral density | 5.40E-10 | 19801982 |
| rs1856057 | 152067869 | Bone mineral density | 3.30E-10 | 19801982 |
| rs1999805* | 152068364 | Bone mineral density | 3.40E-10 | 19801982 |
| rs3020308 | 152068685 | Bone mineral density | 3.70E-10 | 19801982 |
| rs2982575 | 152069791 | Bone mineral density | 8.00E-10 | 19801982 |
| rs2152750 | 152070145 | Bone mineral density | 3.20E-10 | 19801982 |
| rs2982567 | 152075487 | Bone mineral density | 2.90E-10 | 19801982 |
| rs11155811 | 152077846 | Bone mineral density | 7.40E-10 | 19801982 |
| rs1124674 | 152080735 | Bone mineral density | 1.00E-10 | 19801982 |
| rs2504071 | 152084862 | Bone mineral density | 1.50E-10 | 19801982 |
| rs1890010 | 152085275 | Bone mineral density | 9.50E-09 | 19801982 |
| rs2504069 | 152085517 | Bone mineral density | 9.50E-09 | 19801982 |
| rs2504063* | 152090707 | Bone mineral density | 6.10E-11 | 20096396 |
| rs1415193 | 152092638 | Bone mineral density | 3.50E-09 | 19801982 |
| rs543650* | 152110943 | Height | 1.20E-17 | 20881960 |
| rs488133* | 152125444 | Height | 1.20E-10 | 21194676 |
| rs827423 | 152156197 | Height | 3.40E-09 | 20881960 |
| rs827421 | 152157122 | Height | 3.00E-09 | 20881960 |
| rs3853250 | 152159900 | Height | 6.40E-09 | 20881960 |
| rs9397448 | 152161066 | Height | 7.30E-09 | 20881960 |
| rs4870056 | 152162227 | Height | 2.90E-09 | 20881960 |
| rs2234693 | 152163335 | Height | 9.40E-09 | 20881960 |
| rs7739085 | 152164548 | Height | 5.00E-09 | 20881960 |
| rs9322332 | 152166801 | Height | 4.40E-09 | 20881960 |
| rs9479130 | 152168456 | Height | 3.40E-09 | 20881960 |
| rs2982694 | 152285687 | Sudden cardiac arrest | 3.90E-12 | 21658281 |
| rs9340996 | 152342788 | Lipid level measurements | 2.90E-11 | 23063622 |
| rs3020418 | 152345162 | Height | 7.10E-09 | 20881960 |
| rs2982712 | 152358179 | Anthropometric traits | 3.70E-10 | 23563607 |

**Table B**: *ESR1* variants identified through the GRASP catalog of Genome-Wide SNP-Phenotype associations([56](#_ENREF_56)) at a p-value < 1 x 10^-9^. Pubmed IDs for the relevant studies are listed in the rightmost column. Variants marked with “*” have more than one significant association, but only the strongest associations for each variant were reported.

|  | **rs851984** | **rs1285057** | **rs543650** | **rs488133** | **rs2071454** | **TA repeat** | **rs2077647** | **rs2234693** | **rs9340799** | **rs988328** | **rs1801132** | **rs3020327** | **rs3020329** | **rs2144025** |  |
| --- | --- | --- | --- | --- | --- | --- | --- | --- | --- | --- | --- | --- | --- | --- | --- |
| rs1285057 | **0.43** |  |  |  |  |  |  |  |  |  |  |  |  |  | rs1285057 |
|  | *0.07* |  |  |  |  |  |  |  |  |  |  |  |  |  |  |
| rs543650 | **0.16** | **0.41** |  |  |  |  |  |  |  |  |  |  |  |  | rs543650 |
|  | *0.01* | *0.06* |  |  |  |  |  |  |  |  |  |  |  |  |  |
| rs488133 | **0.17** | **0.15** | **0.93** |  |  |  |  |  |  |  |  |  |  |  | rs488133 |
|  | *0.01* | *0.01* | *0.66* |  |  |  |  |  |  |  |  |  |  |  |  |
| rs2071454 | **0.63** | **0.59** | **1.00** | **0.98** |  |  |  |  |  |  |  |  |  |  | rs2071454 |
|  | *0.03* | *0.08* | *0.08* | *0.06* |  |  |  |  |  |  |  |  |  |  |  |
| TA repeat | **0.08** | **0.41** | **0.63** | **0.75** | **0.78** |  |  |  |  |  |  |  |  |  | TA repeat |
|  | *0.00* | *0.08* | *0.28* | *0.30* | *0.07* |  |  |  |  |  |  |  |  |  |  |
| rs2077647 | **0.00** | **0.56** | **0.93** | **0.89** | **1.00** | **0.86** |  |  |  |  |  |  |  |  | rs2077647 |
|  | *0.00* | *0.18* | *0.50* | *0.37* | *0.14* | *0..62* |  |  |  |  |  |  |  |  |  |
| rs2234693 | **0.03** | **0.46** | **0.80** | **0.88** | **0.83** | **0.67** | **0.68** |  |  |  |  |  |  |  | rs2234693 |
|  | *0.00* | *0.13* | *0.36* | *0.34* | *0.10* | *0.36* | *0.46* |  |  |  |  |  |  |  |  |
| rs9340799 | **0.17** | **0.36** | **0.94** | **0.88** | **1.00** | **0.71** | **0.71** | **0.89** |  |  |  |  |  |  | rs9340799 |
|  | *0.01* | *0.12* | *0.31* | *0.19* | *0.08* | *0.24* | *0.30* | *0.47* |  |  |  |  |  |  |  |
| rs988328 | **0.14** | **0.01** | **0.10** | **0.22** | **0.29** | **0.01** | **0.04** | **0.29** | **0.33** |  |  |  |  |  | rs988328 |
|  | *0.01* | *0.00* | *0.00* | *0.02* | *0.07* | *0.00* | *0.00* | *0.01* | *0.01* |  |  |  |  |  |  |
| rs1801132 | **0.54** | **0.02** | **0.14** | **0.10** | **0.23** | **0.05** | **0.17** | **0.24** | **0.49** | **0.85** |  |  |  |  | rs1801132 |
|  | *0..05* | *0.00* | *0.01* | *0.01* | *0.03* | *0.00* | *0.01* | *0.02* | *0.04* | *0.44* |  |  |  |  |  |
| rs3020327 | **0.01** | **0.22** | **0.33** | **0.96** | **0.15** | **0.00** | **0.15** | **0.14** | **0.05** | **0.49** | **0.54** |  |  |  | rs3020327 |
|  | *0.00* | *0.01* | *0.01* | *0.05* | *0.02* | *0.00* | *0.00* | *0.00* | *0.00* | *0.17* | *0.13* |  |  |  |  |
| rs3020329 | **0.30** | **0.16** | **0.18** | **0.24** | **0.19** | **0.15** | **0.20** | **0.11** | **0.01** | **0.41** | **0.25** | **0.77** |  |  | rs3020329 |
|  | *0.02* | *0.02* | *0.01* | *0.01* | *0.01* | *0.01* | *0.02* | *0.00* | *0.00* | *0.08* | *0.05* | *0.20* |  |  |  |
| rs2144025 | **0.25** | **0.22** | **0.50** | **0.61** | **0.34** | **0.01** | **0.24** | **0.11** | **0.11** | **0.51** | **0.46** | **0.88** | **0.76** |  | rs2144025 |
|  | *0.01* | *0.02* | *0.03* | *0.04* | *0.07* | *0.00* | *0.01* | *0.00* | *0.00* | *0.21* | *0.17* | *0.48* | *0.33* |  |  |
| rs3798577 | **0.03** | **0.26** | **0.10** | **0.12** | **0.25** | **0.02** | **0.02** | **0.00** | **0.01** | **0.10** | **0.09** | **0.29** | **0.10** | **0.14** | rs3798577 |
|  | *0.00* | *0.04* | *0.01* | *0.01* | *0.01* | *0.00* | *0.00* | *0.00* | *0.00* | *0.00* | *0.00* | *0.01* | *0.00* | *0.00* |  |
|  | **rs851984** | **rs1285057** | **rs543650** | **rs488133** | **rs2071454** | **TA repeat** | **rs2077647** | **rs2234693** | **rs9340799** | **rs988328** | **rs1801132** | **rs3020327** | **rs3020329** | **rs2144025** |  |

**Table C**: Linkage disequilibrium (LD) of *ESR1* SNPs in the Stanley PFC tissues. Upper values are D’ while the lower values show LD correlation R squared.

Table D: Promoter and Intron4 variants identified by targeted amplicon sequencing. The first 6 variants are in the promoter. Chi-squared p-values and ANODEV P (analysis of deviance) reflect association with AEI (ratios >2) in the 9 sequenced samples. Variants without rs numbers are listed by RefSeqGene position (pos). The RefSeqGene ID was NG_008493.

**Table E:** Allele frequencies, location, and basic allele tests of all *ESR1* SNPs from the GAIN Bipolar Disorder data set, showing the association with number of hypomania episodes. Analyses were conducted with all subjects, and with males and females separately. rs2144025 stands out as most significant among females. Two highly significant scores among males are compromised by the low MAF in this cohort**.**

**Table F:** Association of rs2144025 and other GWAS SNPs across the *ESR1* locus with the presence of grandiose delusions in the GAIN Schizophrenia GWAS.

**Table G**: Association data for rs2144025 and other GWAS SNPs across the *ESR1* locus in the GAIN ADHD GWAS for ADHD subjects with comorbid psychological diagnoses.

| Childhood psychiatric diagnosis RSID | Position | Minor Allele Frequency | Basic allele test P | Dominant allele test P |
| --- | --- | --- | --- | --- |
| rs851972 | 151975773 | 37% | 0.9 | 0.54 |
| rs10484920 | 151979047 | 6% | 0.28 | 0.27 |
| rs9397441 | 151982315 | 10% | 0.58 | 0.61 |
| rs866457 | 151982413 | 43% | 0.47 | 0.32 |
| rs9371226 | 151986594 | 10% | 0.46 | 0.52 |
| rs1293955 | 151990954 | 26% | 0.72 | 0.92 |
| rs1293944 | 151997546 | 48% | 0.18 | 0.11 |
| rs1293940 | 151999091 | 48% | 0.18 | 0.11 |
| rs851995 | 152005534 | 49% | 0.12 | 0.11 |
| rs851993 | 152006011 | 40% | 0.2 | 0.14 |
| rs3020332 | 152008924 | 41% | 0.46 | 0.14 |
| rs2941740 | 152009638 | 39% | 0.65 | 0.27 |
| rs10214867 | 152009654 | 11% | 0.44 | 0.5 |
| rs851996 | 152016803 | 38% | 0.34 | 0.05 |
| rs851985 | 152020390 | 35% | 0.35 | 0.14 |
| rs2347637 | 152028479 | 17% | 0.42 | 0.37 |
| rs851975 | 152031303 | 21% | 0.91 | 0.76 |
| rs12525163 | 152040291 | 23% | 0.77 | 0.48 |
| rs2982565 | 152051854 | 17% | 0.6 | 0.81 |
| rs2982562 | 152052601 | 48% | 0.09 | 9.78E-03 |
| rs3020343 | 152054363 | 49% | 0.06 | 5.91E-03 |
| rs2982560 | 152055606 | 50% | 0.05 | 5.91E-03 |
| rs3020345 | 152056368 | 50% | 0.05 | 5.91E-03 |
| rs2982557 | 152056369 | 50% | 0.05 | 5.91E-03 |
| rs3020346 | 152056771 | 50% | 0.04 | 0.68 |
| rs2982556 | 152056842 | 48% | 0.08 | 6.37E-03 |
| rs2982554 | 152058010 | 47% | 0.03 | 0.54 |
| rs2982552 | 152059563 | 48% | 0.08 | 7.85E-03 |
| rs2982551 | 152061210 | 49% | 0.06 | 4.39E-03 |
| rs3020301 | 152061579 | 50% | 0.05 | 3.20E-03 |
| rs1415194 | 152063998 | 48% | 0.02 | 0.58 |
| rs1999807 | 152064199 | 48% | 0.03 | 0.62 |
| rs3020303 | 152064454 | 48% | 0.03 | 0.62 |
| rs3020304 | 152064464 | 49% | 0.06 | 2.30E-03 |
| rs3020305 | 152064487 | 48% | 0.02 | 0.58 |
| rs3020306 | 152065886 | 48% | 0.08 | 4.74E-03 |
| rs1856057 | 152067869 | 48% | 0.02 | 0.58 |
| rs1999805 | 152068364 | 48% | 7.78E-03 | 0.36 |
| rs2982575 | 152069791 | 50% | 0.04 | 0.78 |
| rs2152750 | 152070145 | 48% | 0.02 | 0.58 |
| rs1361024 | 152070928 | 5% | 0.11 | 0.33 |
| rs1124674 | 152080735 | 43% | 0.18 | 0.51 |
| rs2504071 | 152084862 | 48% | 0.1 | 0.41 |
| rs2504069 | 152085517 | 31% | 0.27 | 0.12 |
| rs17081662 | 152086685 | 2% | 0.36 | 0.36 |
| rs2485209 | 152089768 | 49% | 0.13 | 0.97 |
| rs4870053 | 152092749 | 26% | 0.7 | 0.46 |
| rs7767143 | 152095694 | 21% | 0.5 | 0.38 |
| rs17828471 | 152097405 | 8% | 0.75 | 0.74 |
| rs17081679 | 152101823 | 6% | 0.64 | 0.63 |
| rs528529 | 152102939 | 48% | 0.67 | 0.35 |
| rs11299395 | 152106139 | 4% | 0.62 | 0.61 |
| rs17755779 | 152115403 | 2% | 0.09 | 0.09 |
| rs9478243 | 152117458 | 5% | 0.15 | 0.41 |
| rs9479117 | 152117685 | 15% | 0.39 | 0.78 |
| rs538098 | 152120869 | 3% | 0.48 | 0.47 |
| rs9478244 | 152122037 | 18% | 0.51 | 0.91 |
| rs58202367 | 152122291 | 0% | 0.68 | 0.68 |
| rs17828760 | 152123716 | 3% | 0.25 | 0.24 |
| rs6903180 | 152125231 | 3% | 0.9 | 0.89 |
| rs488133 | 152125444 | 35% | 0.9 | 0.94 |
| rs2077647 | 152129077 | 50% | 0.74 | 0.18 |
| rs576330 | 152132175 | 3% | 0.59 | 0.59 |
| rs10484922 | 152132317 | 9% | 0.92 | 0.85 |
| rs34978802 | 152132431 | 2% | 0.36 | 0.35 |
| rs73780871 | 152147597 | 0% | 0.68 | 0.68 |
| rs7759411 | 152148870 | 2% | 0.99 | 0.99 |
| rs11969288 | 152149200 | 0% | 0.68 | 0.68 |
| rs62442038 | 152149586 | 5% | 0.11 | 0.11 |
| rs12665044 | 152149872 | 12% | 0.97 | 0.97 |
| rs36120076 | 152151566 | 9% | 0.91 | 0.84 |
| rs7761133 | 152151863 | 14% | 0.98 | 0.97 |
| rs17761320 | 152151965 | 5% | 0.92 | 0.92 |
| rs6937568 | 152153964 | 1% | 0.53 | 0.53 |
| rs6902771 | 152157881 | 48% | 0.5 | 0.63 |
| rs62442039 | 152158090 | 4% | 0.55 | 0.61 |
| rs2234693 | 152163335 | 47% | 0.53 | 0.59 |
| rs9340799 | 152163381 | 38% | 0.54 | 0.85 |
| rs7774230 | 152164239 | 47% | 0.57 | 0.64 |
| rs3936674 | 152167311 | 35% | 0.6 | 0.89 |
| rs1709182 | 152175357 | 37% | 0.55 | 0.99 |
| rs827420 | 152177529 | 41% | 0.66 | 0.87 |
| rs827419 | 152177663 | 38% | 0.75 | 0.88 |
| rs9479134 | 152188995 | 2% | 0.27 | 0.27 |
| rs5880948 | 152190236 | 8% | 0.44 | 0.46 |
| rs62443560 | 152190476 | 9% | 0.69 | 0.73 |
| rs9340835 | 152199931 | 35% | 0.64 | 0.48 |
| rs9322334 | 152200041 | 24% | 0.95 | 0.89 |
| rs9322335 | 152200129 | 24% | 0.91 | 0.78 |
| rs9322336 | 152200430 | 22% | 0.9 | 0.77 |
| rs4986934 | 152201875 | 4% | 0.16 | 0.15 |
| rs11155820 | 152204210 | 27% | 0.67 | 0.95 |
| rs4870059 | 152223532 | 2% | 0.28 | 0.27 |
| rs71575914 | 152227990 | 5% | 0.01 | 0.06 |
| rs1514347 | 152229445 | 32% | 0.85 | 0.94 |
| rs12204714 | 152235339 | 44% | 0.22 | 0.28 |
| rs9322343 | 152237759 | 3% | 0.37 | 0.36 |
| rs988328 | 152241150 | 21% | 0.69 | 0.31 |
| rs9479143 | 152245465 | 38% | 0.75 | 0.29 |
| rs9397456 | 152247152 | 31% | 0.62 | 0.11 |
| rs7739274 | 152258509 | 3% | 0.79 | 0.79 |
| rs6912184 | 152260206 | 31% | 0.77 | 0.46 |
| rs4363047 | 152262834 | 31% | 0.77 | 0.46 |
| rs9340894 | 152263380 | 8% | 0.29 | 0.27 |
| rs73005959 | 152270034 | 3% | 0.19 | 0.18 |
| rs35365822 | 152270364 | 15% | 0.57 | 0.68 |
| rs3020314 | 152270672 | 42% | 0.86 | 0.41 |
| rs7745370 | 152274260 | 16% | 0.46 | 0.54 |
| rs3020393 | 152278885 | 23% | 0.65 | 0.48 |
| rs3003921 | 152279514 | 27% | 0.61 | 0.34 |
| rs3020396 | 152279878 | 41% | 0.95 | 0.65 |
| rs1884051 | 152283279 | 41% | 0.99 | 0.6 |
| rs985192 | 152283478 | 26% | 0.66 | 0.38 |
| rs3003925 | 152284458 | 25% | 0.32 | 0.31 |
| rs6557177 | 152284821 | 17% | 0.38 | 0.4 |
| rs985694 | 152286625 | 23% | 0.4 | 0.25 |
| rs9340917 | 152287224 | 2% | 0.7 | 0.7 |
| rs1884049 | 152287367 | 23% | 0.4 | 0.25 |
| rs3020318 | 152289770 | 40% | 0.94 | 0.65 |
| rs73009815 | 152298397 | 2% | 0.32 | 0.31 |
| rs726281 | 152302578 | 31% | 0.13 | 0.08 |
| rs728524 | 152303437 | 2% | 0.17 | 0.17 |
| rs9397463 | 152304328 | 14% | 0.71 | 0.79 |
| rs926777 | 152305047 | 28% | 0.22 | 0.09 |
| rs9371236 | 152306346 | 2% | 0.85 | 0.85 |
| rs3020407 | 152307261 | 35% | 0.18 | 0.06 |
| **rs2144025** | **152307706** | **16%** | **6.20E-03** | **8.96E-04** |
| rs7743290 | 152309132 | 30% | 6.68E-03 | 0.07 |
| rs9340944 | 152313718 | 17% | 0.77 | 0.98 |
| rs722208 | 152322885 | 34% | 2.05E-03 | 0.05 |
| rs13216134 | 152328484 | 13% | 4.43E-05 | 3.64E-05 |
| rs9340955 | 152330201 | 2% | 0.1 | 0.09 |
| rs9340978 | 152333945 | 10% | 0.84 | 0.83 |
| rs9340994 | 152342713 | 8% | 0.16 | 0.18 |
| rs73009834 | 152344346 | 4% | 0.08 | 0.07 |
| rs2982701 | 152347010 | 33% | 0.04 | 0.16 |
| rs9478265 | 152348901 | 6% | 0.37 | 0.35 |
| rs926778 | 152355782 | 32% | 0.02 | 0.12 |
| rs2982712 | 152358179 | 45% | 0.05 | 0.52 |
| rs3020434 | 152358940 | 18% | 0.79 | 0.87 |
| rs2982720 | 152360650 | 38% | 6.58E-03 | 0.14 |
| rs3020368 | 152371190 | 13% | 0.2 | 0.18 |
| rs6913408 | 152378112 | 3% | 0.22 | 0.24 |
| rs9322354 | 152382014 | 14% | 7.05E-04 | 4.96E-04 |
| rs9341019 | 152382688 | 2% | 0.99 | 0.99 |
| rs72993651 | 152385568 | 3% | 0.37 | 0.36 |
| rs73781083 | 152388479 | 3% | 0.25 | 0.27 |
| rs9479193 | 152394779 | 14% | 4.83E-04 | 3.32E-04 |
| rs2747645 | 152396352 | 4% | 0.15 | 0.14 |
| rs2982896 | 152399493 | 23% | 0.8 | 0.61 |
| rs66465244 | 152407061 | 11% | 9.45E-06 | 9.60E-06 |
| rs3778092 | 152408273 | 11% | 9.45E-06 | 9.60E-06 |
| rs2982900 | 152414992 | 10% | 0.54 | 0.57 |
| rs9341056 | 152417142 | 3% | 0.79 | 0.79 |
| rs9341062 | 152419079 | 4% | 0.56 | 0.55 |
| rs3798577 | 152421130 | 46% | 0.27 | 0.53 |
| rs3798758 | 152421854 | 4% | 0.27 | 0.26 |
| rs2747648 | 152422335 | 5% | 0.97 | 0.84 |
| rs9341077 | 152423128 | 5% | 0.58 | 0.57 |
| rs9341086 | 152424534 | 4% | 0.27 | 0.26 |
| rs910416 | 152432902 | 49% | 0.42 | 0.9 |
| rs34133739 | 152434278 | 48% | 0.27 | 0.57 |
| rs73781642 | 152436453 | 3% | 0.48 | 0.47 |
| rs7450824 | 152438103 | 18% | 0.59 | 0.41 |
| rs2813549 | 152441239 | 21% | 0.89 | 0.87 |
| rs11970277 | 152441660 | 3% | 0.48 | 0.47 |
| rs2813554 | 152442338 | 24% | 0.91 | 0.79 |
| rs2250122 | 152443468 | 26% | 0.16 | 0.19 |
| rs2295190 | 152443744 | 9% | 0.3 | 0.3 |
| rs9383964 | 152444815 | 8% | 0.14 | 0.12 |
| rs2747654 | 152447099 | 24% | 0.91 | 0.79 |
| rs2747655 | 152447321 | 21% | 0.96 | 0.96 |
| rs9397486 | 152449900 | 34% | 0.44 | 0.44 |
| rs2459111 | 152450470 | 29% | 0.27 | 0.22 |
| rs6925149 | 152452759 | 5% | 0.2 | 0.19 |

**Table H:** Association data for rs2144025 and other GWAS SNPs across the *ES1* locus in the eMERGE Boston Children’s Hospital study of children and adolescents with comorbid psychological diagnoses. Allele frequencies, location, and basic allele tests of *ESR1* SNPs from The Gene Partnership (TGP) - eMERGE data set, testing an association with any diagnosed psychiatric disease in Caucasian females age 9 or older.

| SNP | Proxy | Distance | RSquared | DPrime | CEU MAF | Coordinate HG18 |
| --- | --- | --- | --- | --- | --- | --- |
| rs2144025 | rs2144025 | 0 | 1 | 1 | 0.125 | 152349399 |
| rs2144025 | rs932477 | 3110 | 0.27 | 1 | 0.033 | 152346289 |
| rs2144025 | rs2179922 | 10606 | 0.27 | 1 | 0.033 | 152338793 |
| rs2144025 | rs9397074 | 22009 | 0.27 | 1 | 0.031 | 152371408 |
| rs2144025 | rs6932902 | 68818 | 0.22 | 0.56 | 0.067 | 152418217 |
| rs2144025 | rs9397080 | 72809 | 0.22 | 0.56 | 0.067 | 152422208 |
| rs2144025 | rs2273206 | 74605 | 0.22 | 0.56 | 0.075 | 152424004 |
| rs2144025 | rs974276 | 74714 | 0.22 | 0.56 | 0.083 | 152424113 |
| rs2144025 | rs985694 | 21081 | 0.20 | 0.51 | 0.150 | 152328318 |

**Table I**: LD and genomic coordinates for rs2144025 and *ESR1* SNPs with clinical associations and with D’ above 0.5 from SNAP ([57](#_ENREF_57)).

| **PWM** | **Strand** | **Ref** | **Alt** | **Match on:**  Ref: GAACAAAATGCACATTTTGTGGACCAACT**T**GTTGTTTTAGATCTATTTTTGAAAACTCA Alt: GAACAAAATGCACATTTTGTGGACCAACT**C**GTTGTTTTAGATCTATTTTTGAAAACTCA |
| --- | --- | --- | --- | --- |
| SIX5_disc4 | - | 7.4 | -4.5 | W**T**GTAGTTTT |
| Zfp105 | - | 11.9 | 7.8 | HNHW**T**KTTDWTTRHD |

**Table J:** Regulatory DNA binding motifs altered by SNP rs2144025, generated by Haploreg([58](#_ENREF_58)).


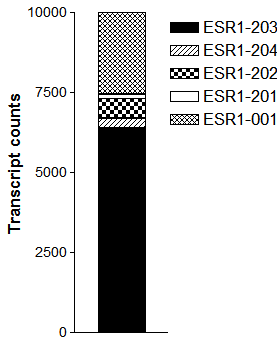

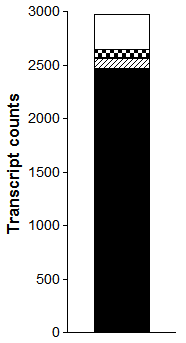

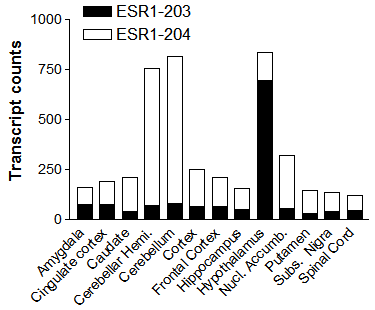

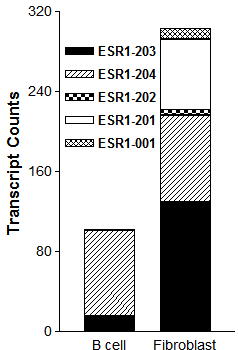


**Liver**

**Breast**

ESR1-203

ESR1-008

ESR1-202

ESR1-201

ESR1-001

ESR1-203

ESR1-008

Fig A: Expression of isoforms in brain and other tissues from the GTEx dataset.

Fig B: Absolute allelic mRNA ratios measured with rs3798577 and rs1801132 in different tissues. Subjects heterozygous for rs2144025 are designated with an asterisk. A dotted line in each graph marks the cut-off ratio (1.4) determining a finding of AEI. Allelic ratios tended to be lower in these tissues than in the Stanley cohort tissues, with only a few just exceeding 2, detected with rs1801132, comparable to allelic ratios in the control group in Fig. 1B. In the brain tissues from the Miami Dade County Brain Bank, 15%of subjects were heterozygous for rs2144025, also comparable to the control group in Fig. 1B.While AEI was detectable, it was not significantly associated with rs2144025


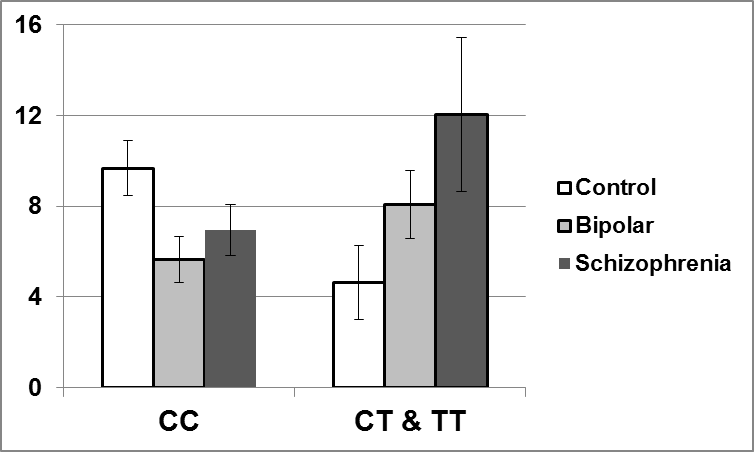

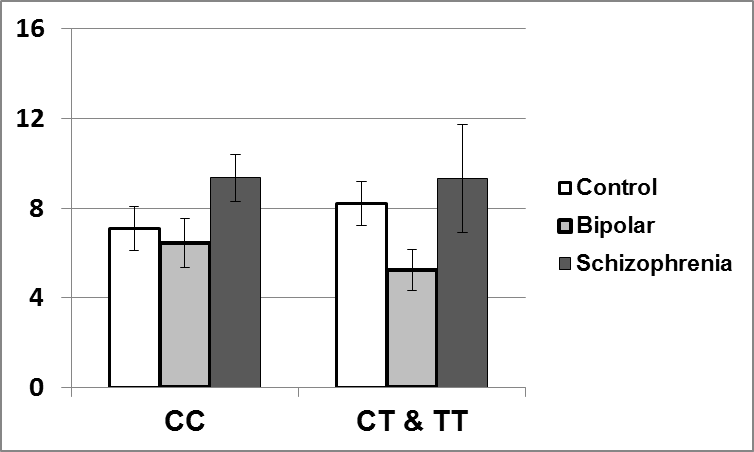


**ESR1-203**

**ESR1-008**

*

*

**Fig C:** Real-time PCR assays of ESR1 mRNA in PFC tissues from the Stanley collection. qRT-PCR was performed employing primers for *beta-actin* and the 3’UTR

(ESR1-203) and exon4 of *ESR1* mRNA, and cycle threshold differences determined(ΔCt).Values Shown are normalized relative levels (linear scale), mean ± SD.

Upper panel: (*) p=0.02, *CC versus CT* controls;; lower panel: *: P=0.03,*CT & TT* BP *versus CT & TT* schizophrenia

Fig D: Top scoring RNA folding structure of 650 base pairs surrounding rs2144025*C>T* was conducted *in silico* using Mfold software. In each structure the position of the SNP is designated by a red arrow (“U” in the left structure and “C” in the right structure).

Supplemental References

1 Rivadeneira, F., Styrkarsdottir, U., Estrada, K., Halldorsson, B.V., Hsu, Y.H., Richards, J.B., Zillikens, M.C., Kavvoura, F.K., Amin, N., Aulchenko, Y.S. *et al.* (2009) Twenty bone-mineral-density loci identified by large-scale meta-analysis of genome-wide association studies. *Nat. Genet.*, **41**, 1199-1206.

2 May, A., Pettifor, J.M., Norris, S.A., Ramsay, M. and Lombard, Z. (2013) Genetic factors influencing bone mineral content in a black South African population. *J. Bone Miner. Metab.*, **31**, 708-716.

3 Tamura, M., Nakayama, T., Sato, I., Sato, N., Izawa, N., Hishiki, M., Mizutani, Y., Furuya, K. and Yamamoto, T. (2008) Haplotype-based case-control study of estrogen receptor alpha (ESR1) gene and pregnancy-induced hypertension. *Hypertens. Res.*, **31**, 221-228.

4 Keene, K.L., Mychaleckyj, J.C., Smith, S.G., Leak, T.S., Perlegas, P.S., Langefeld, C.D., Herrington, D.M., Freedman, B.I., Rich, S.S., Bowden, D.W. *et al.* (2008) Comprehensive evaluation of the estrogen receptor alpha gene reveals further evidence for association with type 2 diabetes enriched for nephropathy in an African American population. *Hum. Genet.*, **123**, 333-341.

5 Alonso, P., Gratacos, M., Segalas, C., Escaramis, G., Real, E., Bayes, M., Labad, J., Pertusa, A., Vallejo, J., Estivill, X. *et al.* (2011) Variants in estrogen receptor alpha gene are associated with phenotypical expression of obsessive-compulsive disorder. *Psychoneuroendocrinology*, **36**, 473-483.

6 Pinsonneault, J.K., Sullivan, D., Sadee, W., Soares, C.N., Hampson, E. and Steiner, M. (2013) Association study of the estrogen receptor gene ESR1 with postpartum depression--a pilot study. *Arch Womens Ment Health*, **16**, 499-509.

7 Tang, S., Yue, M., Wang, J., Su, J., Yu, R., Zhou, D., Xu, K., Cai, L. and Zhang, Y. (2014) Association of genetic variants in estrogen receptor alpha with HCV infection susceptibility and viral clearance in a high-risk Chinese population. *Eur. J. Clin. Microbiol. Infect. Dis.*, in press.

8 Schupf, N., Lee, J.H., Wei, M., Pang, D., Chace, C., Cheng, R., Zigman, W.B., Tycko, B. and Silverman, W. (2008) Estrogen receptor-alpha variants increase risk of Alzheimer's disease in women with Down syndrome. *Dement. Geriatr. Cogn. Disord.*, **25**, 476-482.

9 Sonoda, T., Suzuki, H., Mori, M., Tsukamoto, T., Yokomizo, A., Naito, S., Fujimoto, K., Hirao, Y., Miyanaga, N. and Akaza, H. (2010) Polymorphisms in estrogen related genes may modify the protective effect of isoflavones against prostate cancer risk in Japanese men. *Eur. J. Cancer. Prev.*, **19**, 131-137.

10 Mill, J., Kiss, E., Baji, I., Kapornai, K., Daróczy, G., Vetró, A., Kennedy, J., Kovacs, M. and Barr, C. (2008) Association study of the estrogen receptor alpha gene (ESR1) and childhood-onset mood disorders. *Am. J. Med. Genet. B Neuropsychiatr. Genet.*, **147B**, 1323-1326.

11 Sonoda, T., Takada, J., Iba, K., Asakura, S., Yamashita, T. and Mori, M. (2012) Interaction between ESRalpha polymorphisms and environmental factors in osteoporosis. *J. Orthop. Res.*, **30**, 1529-1534.

12 Boada, M., Antunez, C., Lopez-Arrieta, J., Caruz, A., Moreno-Rey, C., Ramirez-Lorca, R., Moron, F.J., Hernandez, I., Mauleon, A., Rosende-Roca, M. *et al.* (2012) Estrogen receptor alpha gene variants are associated with Alzheimer's disease. *Neurobiol. Aging*, **33**, 198 e115-124.

13 Wang, C., Zhang, Z., Zhang, H., He, J.W., Gu, J.M., Hu, W.W., Hu, Y.Q., Li, M., Liu, Y.J., Fu, W.Z. *et al.* (2012) Susceptibility genes for osteoporotic fracture in postmenopausal Chinese women. *J. Bone Miner. Res.*, **27**, 2582-2591.

14 Klos, K.L., Boerwinkle, E., Ferrell, R.E., Turner, S.T. and Morrison, A.C. (2008) ESR1 polymorphism is associated with plasma lipid and apolipoprotein levels in Caucasians of the Rochester Family Heart Study. *J. Lipid Res.*, **49**, 1701-1706.

15 Yaffe, K., Lindquist, K., Sen, S., Cauley, J., Ferrell, R., Penninx, B., Harris, T., Li, R. and Cummings, S.R. (2009) Estrogen receptor genotype and risk of cognitive impairment in elders: findings from the Health ABC study. *Neurobiol. Aging*, **30**, 607-614.

16 Wedren, S., Lovmar, L., Humphreys, K., Magnusson, C., Melhus, H., Syvanen, A.C., Kindmark, A., Landegren, U., Fermer, M.L., Stiger, F. *et al.* (2008) Estrogen receptor alpha gene polymorphism and endometrial cancer risk--a case-control study. *BMC Cancer*, **8**, 322.

17 Kurt, O., Yilmaz-Aydogan, H., Uyar, M., Isbir, T., Seyhan, M.F. and Can, A. (2012) Evaluation of ERalpha and VDR gene polymorphisms in relation to bone mineral density in Turkish postmenopausal women. *Mol. Biol. Rep.*, **39**, 6723-6730.

18 Yan, Z., Tan, W., Xu, B., Dan, Y., Zhao, W., Deng, C., Chen, W., Tan, S., Mao, Q., Wang, Y. *et al.* (2011) A cis-acting regulatory variation of the estrogen receptor alpha (ESR1) gene is associated with hepatitis B virus-related liver cirrhosis. *Hum. Mutat.*, **32**, 1128-1136.

19 Ryan, J., Carriere, I., Carcaillon, L., Dartigues, J.F., Auriacombe, S., Rouaud, O., Berr, C., Ritchie, K., Scarabin, P.Y. and Ancelin, M.L. (2014) Estrogen receptor polymorphisms and incident dementia: The prospective 3C study. *Alzheimers Dement.*, **10**, 27-35.

20 Ryan, J., Scali, J., Carriere, I., Peres, K., Rouaud, O., Scarabin, P.Y., Ritchie, K. and Ancelin, M.L. (2011) Oestrogen receptor polymorphisms and late-life depression. *Br. J. Psychiatry*, **199**, 126-131.

21 Ryan, J., Scali, J., Carriere, I., Scarabin, P.Y., Ritchie, K. and Ancelin, M.L. (2011) Estrogen receptor gene variants are associated with anxiety disorders in older women. *Psychoneuroendocrinology*, **36**, 1582-1586.

22 Dziedziejko, V., Kurzawski, M., Safranow, K., Drozdzik, M., Chlubek, D. and Pawlik, A. (2011) Oestrogen receptor polymorphisms in female patients with rheumatoid arthritis. *Scand. J. Rheumatol.*, **40**, 329-333.

23 Borgonio-Cuadra, V.M., Gonzalez-Huerta, C., Duarte-Salazar, C., de Los Angeles Soria-Bastida, M., Cortes-Gonzalez, S. and Miranda-Duarte, A. (2012) Analysis of estrogen receptor alpha gene haplotype in Mexican mestizo patients with primary osteoarthritis of the knee. *Rheumatol. Int.*, **32**, 1425-1430.

24 Safarinejad, M.R., Safarinejad, S. and Shafiei, N. (2012) Estrogen receptors alpha (rs2234693 and rs9340799), and beta (rs4986938 and rs1256049) genes polymorphism in prostate cancer: evidence for association with risk and histopathological tumor characteristics in Iranian men. *Mol. Carcinog.*, **51 Suppl 1**, E104-117.

25 Zhao, T., Zhang, D., Liu, Y., Zhou, D., Chen, Z., Yang, Y., Li, S., Yu, L., Zhang, Z., Feng, G. *et al.* (2010) Association between ESR1 and ESR2 gene polymorphisms and hyperlipidemia in Chinese Han postmenopausal women. *J. Hum. Genet.*, **55**, 50-54.

26 Peter, I., Kelley-Hedgepeth, A., Huggins, G.S., Housman, D.E., Mendelsohn, M.E., Vita, J.A., Vasan, R.S., Levy, D., Benjamin, E.J. and Mitchell, G.F. (2009) Association between arterial stiffness and variations in oestrogen-related genes. *J. Hum. Hypertens.*, **23**, 636-644.

27 Scacchi, R., Gambina, G., Broggio, E. and Corbo, R.M. (2013) Sex and ESR1 genotype may influence the response to treatment with donepezil and rivastigmine in patients with Alzheimer's disease. *Int. J. Geriatr. Psychiatry.*, in press.

28 Henry, N.L., Skaar, T.C., Dantzer, J., Li, L., Kidwell, K., Gersch, C., Nguyen, A.T., Rae, J.M., Desta, Z., Oesterreich, S. *et al.* (2013) Genetic associations with toxicity-related discontinuation of aromatase inhibitor therapy for breast cancer. *Breast Cancer Res. Treat.*, **138**, 807-816.

29 Kelly, T.N., Rebholz, C.M., Gu, D., Hixson, J.E., Rice, T.K., Cao, J., Chen, J., Li, J., Lu, F., Ma, J. *et al.* (2013) Analysis of sex hormone genes reveals gender differences in the genetic etiology of blood pressure salt sensitivity: the GenSalt study. *Am. J. Hypertens.*, **26**, 191-200.

30 Kaunisto, M.A., Kallela, M., Hamalainen, E., Kilpikari, R., Havanka, H., Harno, H., Nissila, M., Sako, E., Ilmavirta, M., Liukkonen, J. *et al.* (2006) Testing of variants of the MTHFR and ESR1 genes in 1798 Finnish individuals fails to confirm the association with migraine with aura. *Cephalalgia*, **26**, 1462-1472.

31 Roberts, K.E., Fallon, M.B., Krowka, M.J., Brown, R.S., Trotter, J.F., Peter, I., Tighiouart, H., Knowles, J.A., Rabinowitz, D., Benza, R.L. *et al.* (2009) Genetic risk factors for portopulmonary hypertension in patients with advanced liver disease. *Am. J. Respir. Crit. Care Med.*, **179**, 835-842.

32 Sonestedt, E., Ivarsson, M.I., Harlid, S., Ericson, U., Gullberg, B., Carlson, J., Olsson, H., Adlercreutz, H. and Wirfalt, E. (2009) The protective association of high plasma enterolactone with breast cancer is reasonably robust in women with polymorphisms in the estrogen receptor alpha and beta genes. *J. Nutr.*, **139**, 993-1001.

33 Dahlman, I., Vaxillaire, M., Nilsson, M., Lecoeur, C., Gu, H.F., Cavalcanti-Proenca, C., Efendic, S., Ostenson, C.G., Brismar, K., Charpentier, G. *et al.* (2008) Estrogen receptor alpha gene variants associate with type 2 diabetes and fasting plasma glucose. *Pharmacogenet. Genomics*, **18**, 967-975.

34 Velasco, J., Hernandez, J.L., Perez-Castrillon, J.L., Zarrabeitia, M.T., Alonso, M.A., Gonzalez-Macias, J. and Riancho, J.A. (2010) Haplotypes of intron 4 of the estrogen receptor alpha gene and hip fractures: a replication study in Caucasians. *BMC Med. Genet.*, **11**, 16.

35 Dunning, A.M., Healey, C.S., Baynes, C., Maia, A.T., Scollen, S., Vega, A., Rodriguez, R., Barbosa-Morais, N.L., Ponder, B.A., Low, Y.L. *et al.* (2009) Association of ESR1 gene tagging SNPs with breast cancer risk. *Hum. Mol. Genet.*, **18**, 1131-1139.

36 Aouizerat, B.E., Vittinghoff, E., Musone, S.L., Pawlikowska, L., Kwok, P.Y., Olgin, J.E. and Tseng, Z.H. (2011) GWAS for discovery and replication of genetic loci associated with sudden cardiac arrest in patients with coronary artery disease. *BMC Cardiovasc. Disord.*, **11**, 29.

37 Kiel, D.P., Demissie, S., Dupuis, J., Lunetta, K.L., Murabito, J.M. and Karasik, D. (2007) Genome-wide association with bone mass and geometry in the Framingham Heart Study. *BMC Med. Genet.*, **8 Suppl 1**, S14.

38 Dahlgren, A., Lundmark, P., Axelsson, T., Lind, L. and Syvanen, A.C. (2008) Association of the estrogen receptor 1 (ESR1) gene with body height in adult males from two Swedish population cohorts. *PLoS ONE*, **3**, e1807.

39 Versini, A., Ramoz, N., Le Strat, Y., Scherag, S., Ehrlich, S., Boni, C., Hinney, A., Hebebrand, J., Romo, L., Guelfi, J.D. *et al.* (2010) Estrogen receptor 1 gene (ESR1) is associated with restrictive anorexia nervosa. *Neuropsychopharmacology*, **35**, 1818-1825.

40 Einarsdottir, K., Darabi, H., Li, Y., Low, Y.L., Li, Y.Q., Bonnard, C., Sjolander, A., Czene, K., Wedren, S., Liu, E.T. *et al.* (2008) ESR1 and EGF genetic variation in relation to breast cancer risk and survival. *Breast Cancer Res.*, **10**, R15.

41 Ahn, J., Schumacher, F.R., Berndt, S.I., Pfeiffer, R., Albanes, D., Andriole, G.L., Ardanaz, E., Boeing, H., Bueno-de-Mesquita, B., Chanock, S.J. *et al.* (2009) Quantitative trait loci predicting circulating sex steroid hormones in men from the NCI-Breast and Prostate Cancer Cohort Consortium (BPC3). *Hum. Mol. Genet.*, **18**, 3749-3757.

42 Giegling, I., Chiesa, A., Calati, R., Hartmann, A.M., Moller, H.J., De Ronchi, D., Rujescu, D. and Serretti, A. (2009) Do the estrogen receptors 1 gene variants influence the temperament and character inventory scores in suicidal attempters and healthy subjects? *Am. J. Med. Gene.t B Neuropsychiatr. Genet.*, **150B**, 434-438.

43 Hamaguchi, M., Nishio, M., Toyama, T., Sugiura, H., Kondo, N., Fujii, Y. and Yamashita, H. (2008) Possible difference in frequencies of genetic polymorphisms of estrogen receptor alpha, estrogen metabolism and P53 genes between estrogen receptor-positive and -negative breast cancers. *Jpn. J. Clin. Oncol.*, **38**, 734-742.

44 Kim, S., Pyun, J.A., Kang, H., Kim, J., Cha, D.H. and Kwack, K. (2011) Epistasis between CYP19A1 and ESR1 polymorphisms is associated with premature ovarian failure. *Fertil. Steril.*, **95**, 353-356.

45 Romerius, P., Giwercman, A., Moell, C., Relander, T., Cavallin-Stahl, E., Wiebe, T., Hallden, C. and Giwercman, Y.L. (2011) Estrogen receptor alpha single nucleotide polymorphism modifies the risk of azoospermia in childhood cancer survivors. *Pharmacogenet. Genomics*, **21**, 263-269.

46 van der Zanden, L.F., Galesloot, T.E., Feitz, W.F., Brouwers, M.M., Shi, M., Knoers, N.V., Franke, B., Roeleveld, N. and van Rooij, I.A. (2012) Exploration of gene-environment interactions, maternal effects and parent of origin effects in the etiology of hypospadias. *J. Urol.*, **188**, 2354-2360.

47 Brokken, L.J., Lundberg-Giwercman, Y., Rajpert De-Meyts, E., Eberhard, J., Stahl, O., Cohn-Cedermark, G., Daugaard, G., Arver, S. and Giwercman, A. (2012) Association of polymorphisms in genes encoding hormone receptors ESR1, ESR2 and LHCGR with the risk and clinical features of testicular germ cell cancer. *Mol. Cell. Endocrinol.*, **351**, 279-285.

48 Weickert, C., Miranda-Angulo, A., Wong, J., Perlman, W., Ward, S., Radhakrishna, V., Straub, R., Weinberger, D. and Kleinman, J. (2008) Variants in the estrogen receptor alpha gene and its mRNA contribute to risk for schizophrenia. *Hum. Mol. Gen.*, **17**, 2293-2309.

49 Linner, C., Svartberg, J., Giwercman, A. and Giwercman, Y.L. (2013) Estrogen receptor alpha single nucleotide polymorphism as predictor of diabetes type 2 risk in hypogonadal men. *Aging Male*, **16**, 52-57.

50 Galan, J.J., Buch, B., Pedrinaci, S., Jimenez-Gamiz, P., Gonzalez, A., Serrano-Rios, M., Salinas, A., Rivero Mdel, C., Real, L.M., Royo, J.L. *et al.* (2008) Identification of a 2244 base pair interstitial deletion within the human ESR1 gene in the Spanish population. *J. Med. Genet.*, **45**, 420-424.

51 Harlid, S., Ivarsson, M.I., Butt, S., Hussain, S., Grzybowska, E., Eyfjord, J.E., Lenner, P., Forsti, A., Hemminki, K., Manjer, J. *et al.* (2011) A candidate CpG SNP approach identifies a breast cancer associated ESR1-SNP. *Int. J. Cancer*, **129**, 1689-1698.

52 Wu, M.M., Hsieh, Y.C., Lien, L.M., Chen, W.H., Bai, C.H., Chiu, H.C., Chen, H.H., Chung, W.T., Lee, Y.C., Hsu, C.Y. *et al.* (2010) Association of estrogen receptor {alpha} genotypes/ haplotypes with carotid intima-media thickness in Taiwanese women. *Angiology*, **61**, 275-282.

53 Slof-Op 't Landt, M.C., van Furth, E.F., Meulenbelt, I., Bartels, M., Hottenga, J.J., Slagboom, P.E. and Boomsma, D.I. (2013) Association study of the estrogen receptor I gene (ESR1) in anorexia nervosa and eating disorders: No replication found. *Int. J. Eat. Disord.*, in press.

54 Zettergren, A., Jonsson, L., Johansson, D., Melke, J., Lundstrom, S., Anckarsater, H., Lichtenstein, P. and Westberg, L. (2013) Associations between polymorphisms in sex steroid related genes and autistic-like traits. *Psychoneuroendocrinology*, **38**, 2575-2584.

55 Levesque, E., Huang, S.P., Audet-Walsh, E., Lacombe, L., Bao, B.Y., Fradet, Y., Laverdiere, I., Rouleau, M., Huang, C.Y., Yu, C.C. *et al.* (2013) Molecular markers in key steroidogenic pathways, circulating steroid levels, and prostate cancer progression. *Clin. Cancer Res.*, **19**, 699-709.

56 Leslie, R., O'Donnell, C.J. and Johnson, A.D. (2014) GRASP: analysis of genotype-phenotype results from 1390 genome-wide association studies and corresponding open access database. *Bioinformatics (Oxford, England)*, **30**, i185-194.

57 Johnson, A.D., Handsaker, R.E., Pulit, S.L., Nizzari, M.M., O'Donnell, C.J. and de Bakker, P.I. (2008) SNAP: a web-based tool for identification and annotation of proxy SNPs using HapMap. *Bioinformatics*, **24**, 2938-2939.

58 Ward, L.D. and Kellis, M. (2012) HaploReg: a resource for exploring chromatin states, conservation, and regulatory motif alterations within sets of genetically linked variants. *Nucleic acids research*, **40**, D930-934.

**Acknowledgements**

Details of funding sources for the GWAS studies used in this work are as follows.

*Genetic Association Information Network (GAIN) Whole Genome Association of Bipolar Disorder version 3 (Accession: phs000017.v3.p1).*

Funding support for the Whole Genome Association Study of Bipolar Disorder was provided by the National Institute of Mental Health (NIMH) and the genotyping of samples was provided through the Genetic Association Information Network (GAIN). The datasets used for the analyses described in this manuscript were obtained from the database of Genotypes and Phenotypes (dbGaP) found at http://www.ncbi.nlm.nih.gov/gap through dbGaP accession number *phs000017.v3.p1*. Samples and associated phenotype data for the Collaborative Genomic Study of Bipolar Disorder were provided by the The NIMH Genetics Initiative for Bipolar Disorder. Data and biomaterials were collected in four projects that participated in NIMH Bipolar Disorder Genetics Initiative. From 1991-98, the Principal Investigators and Co-Investigators were: Indiana University, Indianapolis, IN, U01 MH46282, John Nurnberger, M.D., Ph.D., Marvin Miller, M.D., and Elizabeth Bowman, M.D.; Washington University, St. Louis, MO, U01 MH46280, Theodore Reich, M.D., Allison Goate, Ph.D., and John Rice, Ph.D.; Johns Hopkins University, Baltimore, MD U01 MH46274, J. Raymond DePaulo, Jr., M.D., Sylvia Simpson, M.D., MPH, and Colin Stine, Ph.D.; NIMH Intramural Research Program, Clinical Neurogenetics Branch, Bethesda, MD, Elliot Gershon, M.D., Diane Kazuba, B.A., and Elizabeth Maxwell, M.S.W. Data and biomaterials were collected as part of ten projects that participated in the NIMH Bipolar Disorder Genetics Initiative. From 1999-03, the Principal Investigators and Co-Investigators were: Indiana University, Indianapolis, IN, R01 MH59545, John Nurnberger, M.D., Ph.D., Marvin J. Miller, M.D., Elizabeth S. Bowman, M.D., N. Leela Rau, M.D., P. Ryan Moe, M.D., NaliniSamavedy, M.D., Rif El-Mallakh, M.D. (at University of Louisville), Husseini Manji, M.D. (at Wayne State University), Debra A. Glitz, M.D. (at Wayne State University), Eric T. Meyer, M.S., Carrie Smiley, R.N., Tatiana Foroud, Ph.D., Leah Flury, M.S., Danielle M. Dick, Ph.D., Howard Edenberg, Ph.D.; Washington University, St. Louis, MO, R01 MH059534, John Rice, Ph.D, Theodore Reich, M.D., Allison Goate, Ph.D., Laura Bierut, M.D. ; Johns Hopkins University, Baltimore, 6 GAIN: Whole Genome Association Study of Bipolar Disorder July 26, 2013 version MD, R01 MH59533, Melvin McInnis M.D. , J. Raymond DePaulo, Jr., M.D., Dean F. MacKinnon, M.D., Francis M. Mondimore, M.D., James B. Potash, M.D., Peter P. Zandi, Ph.D, Dimitrios Avramopoulos, and Jennifer Payne; University of Pennsylvania, PA, R01 MH59553, Wade Berrettini M.D.,Ph.D. ; University of California at Irvine, CA, R01 MH60068, William Byerley M.D., and Mark Vawter M.D. ; University of Iowa, IA, R01 MH059548, William Coryell M.D. , and Raymond Crowe M.D. ; University of Chicago, IL, R01 MH59535, Elliot Gershon, M.D., Judith Badner Ph.D. , Francis McMahon M.D. , Chunyu Liu Ph.D., Alan Sanders M.D., Maria Caserta, Steven Dinwiddie M.D., Tu Nguyen, Donna Harakal; University of California at San Diego, CA, R01 MH59567, John Kelsoe, M.D., Rebecca McKinney, B.A.; Rush University, IL, R01 MH059556, William Scheftner M.D. , Howard M. Kravitz, D.O., M.P.H., Diana Marta, B.S., Annette VaughnBrown, MSN, RN, and Laurie Bederow, MA; NIMH Intramural Research Program, Bethesda, MD, 1Z01MH002810-01, Francis J. McMahon, M.D., Layla Kassem, PsyD, SevillaDetera-Wadleigh, Ph.D, Lisa Austin,Ph.D, Dennis L. Murphy, M.D.

*Genetic Association Information Network (GAIN) Genome-Wide Association Study of Schizophrenia version 3 (Accession: phs000021.v3.p2).*

Funding support for the Genome-Wide Association of Schizophrenia Study was provided by the National Institute of Mental Health (R01 MH67257, R01 MH59588, R01 MH59571, R01 MH59565, R01 MH59587, R01 MH60870, R01 MH59566, R01 MH59586, R01 MH61675, R01 MH60879, R01 MH81800, U01 MH46276, U01 MH46289 U01 MH46318, U01 MH79469, and U01 MH79470) and the genotyping of samples was provided through the Genetic Association Information Network (GAIN). The datasets used for the analyses described in this manuscript were obtained from the database of Genotypes and Phenotypes (dbGaP) found at http://www.ncbi.nlm.nih.gov/gap through dbGaP accession number *phs000021.v3.p2*. Samples and associated phenotype data for the Genome-Wide Association of Schizophrenia Study were provided by the Molecular Genetics of Schizophrenia Collaboration (PI: Pablo V. Gejman, Evanston Northwestern Healthcare (ENH) and Northwestern University, Evanston, IL, USA).

*International Multi-Center ADHD Genetics Project (Accession: phs000016.v2.p2), part of Genetic Association Information Network (GAIN).*

Funding support for the International Multisite ADHD Genetics (IMAGE) project was provided by NIH grants R01MH62873 and R01MH081803 to S.V. Faraone and the genotyping of samples was provided through the Genetic Association Information Network (GAIN). The dataset(s) used for the analyses described in this manuscript were obtained from the database of Genotypes and Phenotypes (dbGaP) found at http://www.ncbi.nlm.nih.gov/gap through dbGaP accession number *phs000016.v2.p2*. Samples and associated phenotype data for the International Multi-Center ADHD Genetics Project were provided by the following investigators: S. Faraone (PI), R. Anney, P. Asherson, J. Sergeant, R. Ebstein, B. Franke, M. Gill, A. Miranda, F. Mulas, R. Oades, H. Roeyers, A. Rothenberger, T. Banaschewski, J. Buitelaar, E. Sonuga-Barke (site PIs), M. Daly, C. Lange, N. Laird, J. Su, and B. Neale (statistical analysis team).

*The Gene Partnership (TGP) - eMERGE Data Distribution Set 1 (Accession:phs000495.v1.p1)*

Samples and data used in this study are provided by The Gene Partnership (TGP) (http://www.genepartnership.org/) a prospective longitudinal study to study the genetic and environmental contributions to childhood health and diseases, collect genetic information on a large number of children who have been phenotyped, and implement the Informed Cohort and the Informed Cohort Oversight Board (ICOB). Children's Hospital Boston (CHB) has committed $10 million for the start-up of the TGP. The datasets used for the analyses described in this manuscript were obtained from dbGaP at http://www.ncbi.nlm.nih.gov/gap through dbGaP accession number phs000495.v1.p1

*The GTEx RNA-Seq and Whole Exome Sequencing data used for analysis in this manuscript were from dbGaP accession number phs000424.v5.p1.*

The Genotype-Tissue Expression (GTEx) Project was supported by the Common Fund of the Office of the Director of the National Institutes of Health (commonfund.nih.gov/GTEx). Additional funds were provided by the NCI, NHGRI, NHLBI, NIDA, NIMH, and NINDS. Donors were enrolled at Biospecimen Source Sites funded by NCI\Leidos Biomedical Research, Inc. subcontracts to the National Disease Research Interchange (10XS170), GTEx Project March 5, 2014 version Page 5 of 8 Roswell Park Cancer Institute (10XS171), and Science Care, Inc. (X10S172). The Laboratory, Data Analysis, and Coordinating Center (LDACC) was funded through a contract (HHSN268201000029C) to the The Broad Institute, Inc. Biorepository operations were funded through a Leidos Biomedical Research, Inc. subcontract to Van Andel Research Institute (10ST1035). Additional data repository and project management were provided by Leidos Biomedical Research, Inc.(HHSN261200800001E). The Brain Bank was supported supplements to University of Miami grant DA006227. Statistical Methods development grants were made to the University of Geneva (MH090941 & MH101814), the University of Chicago (MH090951,MH090937, MH101825, & MH101820), the University of North Carolina - Chapel Hill (MH090936), North Carolina State University (MH101819),Harvard University (MH090948), Stanford University (MH101782), Washington University (MH101810), and to the University of Pennsylvania (MH101822). The datasets used for the analyses described in this manuscript were obtained from dbGaP at http://www.ncbi.nlm.nih.gov/gap through dbGaP accession number *phs000424.v5.p1.*
